# Supplementary material for: Metabolic remodeling in hiPSC-derived myofibers carrying the m.3243A>G mutation
Source: Stem Cell Reports. 2025 Mar 13;20(4):102448. doi: 10.1016/j.stemcr.2025.102448 (PMC12069895; doi:10.1016/j.stemcr.2025.102448)
Supplement: Document S1. Figures S1–S5 and supplemental methods [file mmc1.pdf]

Stem Cell Reports, Volume *20*

Supplemental Information

Metabolic remodeling in hiPSC-derived myofibers carrying the m.3243A>G mutation

Gabriel E. Valdebenito, Anitta R. Chacko, Chih-Yao Chung, Preethi Sheshadri, Haoyu Chi, Benjamin O'Callaghan, Monika J. Madej, Henry Houlden, Hannah Rouse, Valle Morales, Katiuscia Bianchi, Francesco Saverio Tedesco, Robert D.S. Pitceathly, and Michael R. Duchen

SUPPLEMENTAL FIGURES

Fig S1

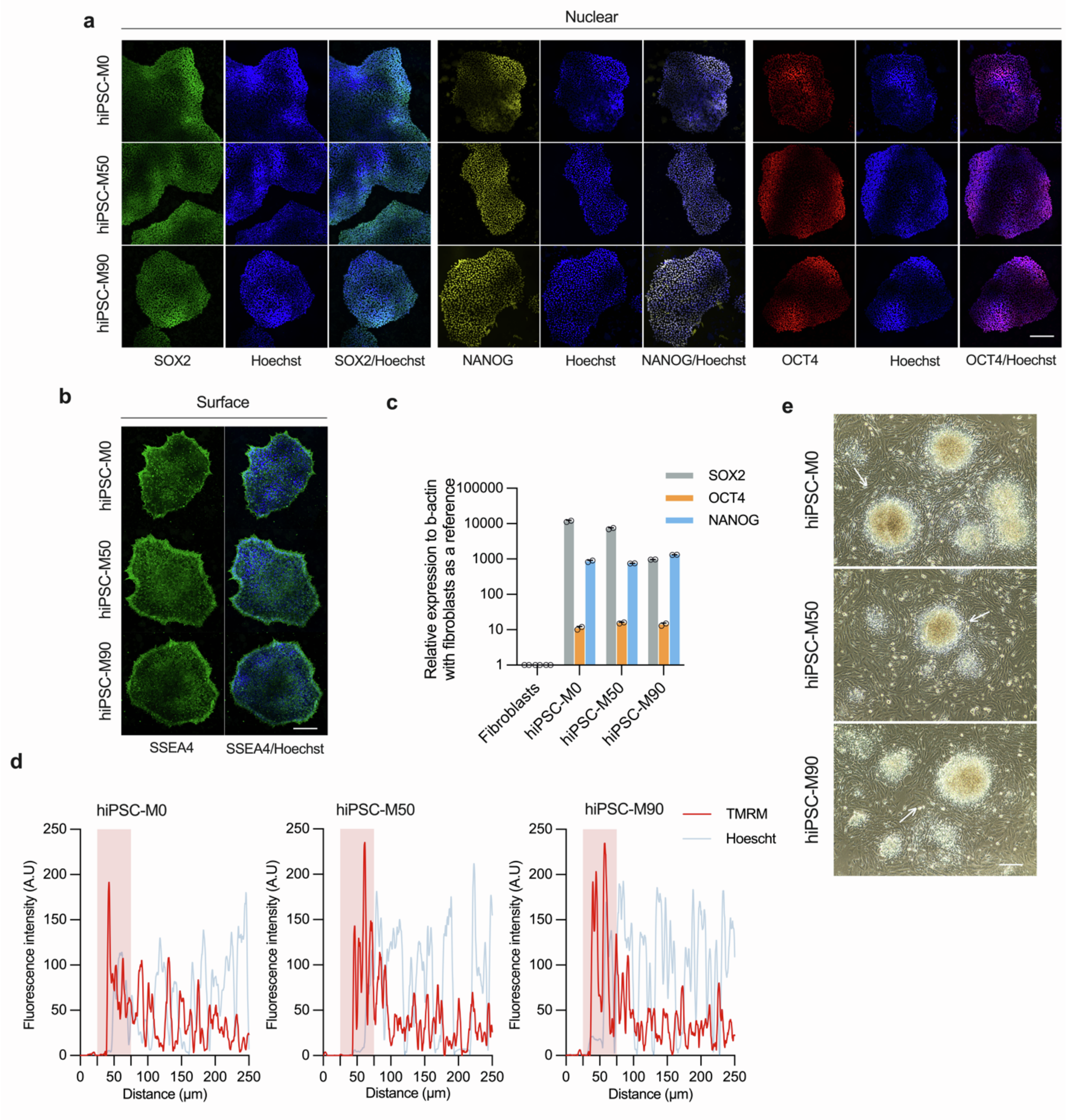

Supplementary Figure 1. Maintenance of pluripotency and mitochondrial function during the undifferentiated stage of hiPS cells.

(A) Representative images of hiPS cell colonies stained for nuclear pluripotency markers SOX2, NANOG and OCT4. Scale bar = 100 µm.

(B) Representative images of hiPS cell colonies stained for the pluripotency surface marker SSEA4. Scale bar = 100 µm.

(C) Relative gene expression of pluripotency markers thought RT-qPCR (*n* = 2 independent biological samples).

(D) Cross sectional fluorescence quantification of hiPSC colonies loaded with 25 nM TMRM. Coloured background represents 50 µM from the border of the colony to an arbitrary centre.

(E) Bright-field images of differentiating cells depicting the formation of myocentres. Arrows indicate the myocentres. Scale bar = 100 µm.

Source data are provided as a Source Data file. All data were represented as mean ± SD.

Fig S2

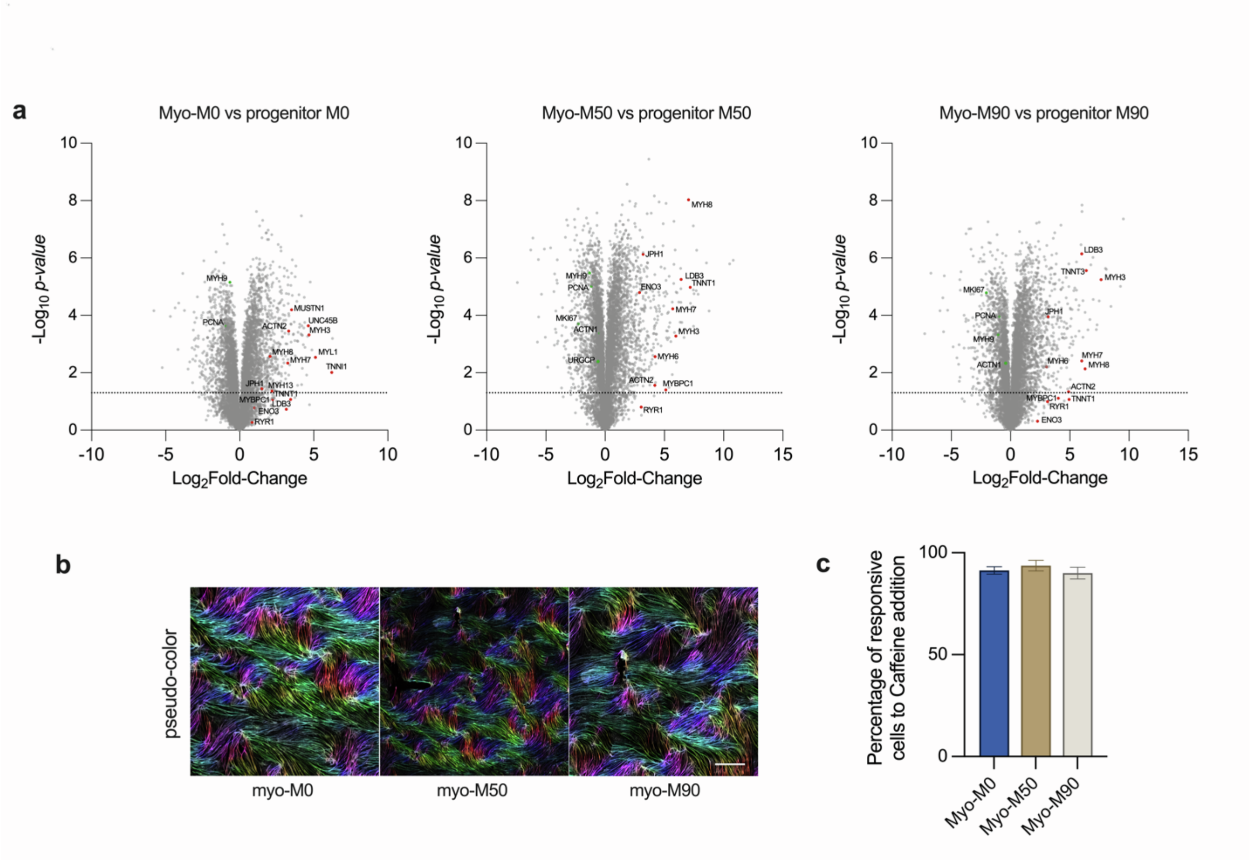

Supplementary Figure 2. Changes in the proteome after differentiation, bundle formation and calcium responses in myofibres derived from hiPSCs.

(A) Volcano plot showing changes in the proteome. Changes are expressed as myofibre over its respective progenitor for each line. Genes above the dotted line express significant changes (-Log p-value ≥ 1.3). Red dots = upregulated proteins; green dots = downregulated proteins.

(B) Representative confocal images of myofibres stained with antibodies against alpha-actinin 2 and pseudo-coloured in relation to the fibre orientation. Scale bar = 500 µm.

(C) Quantification of responsive myofibres to caffeine addition. Arbitrary threshold of 10% change over basal intensity was defined to consider a cell as responsive. n > 100 myofibres were analysed per condition.

Source data are provided as a Source Data file. All data were represented as mean ± SD and data were analysed by one-way ANOVA with Tukey's multiple comparisons test (*p < 0.05, **p < 0.01, ***p < 0.001, ****p < 0.

0001).

Fig S3

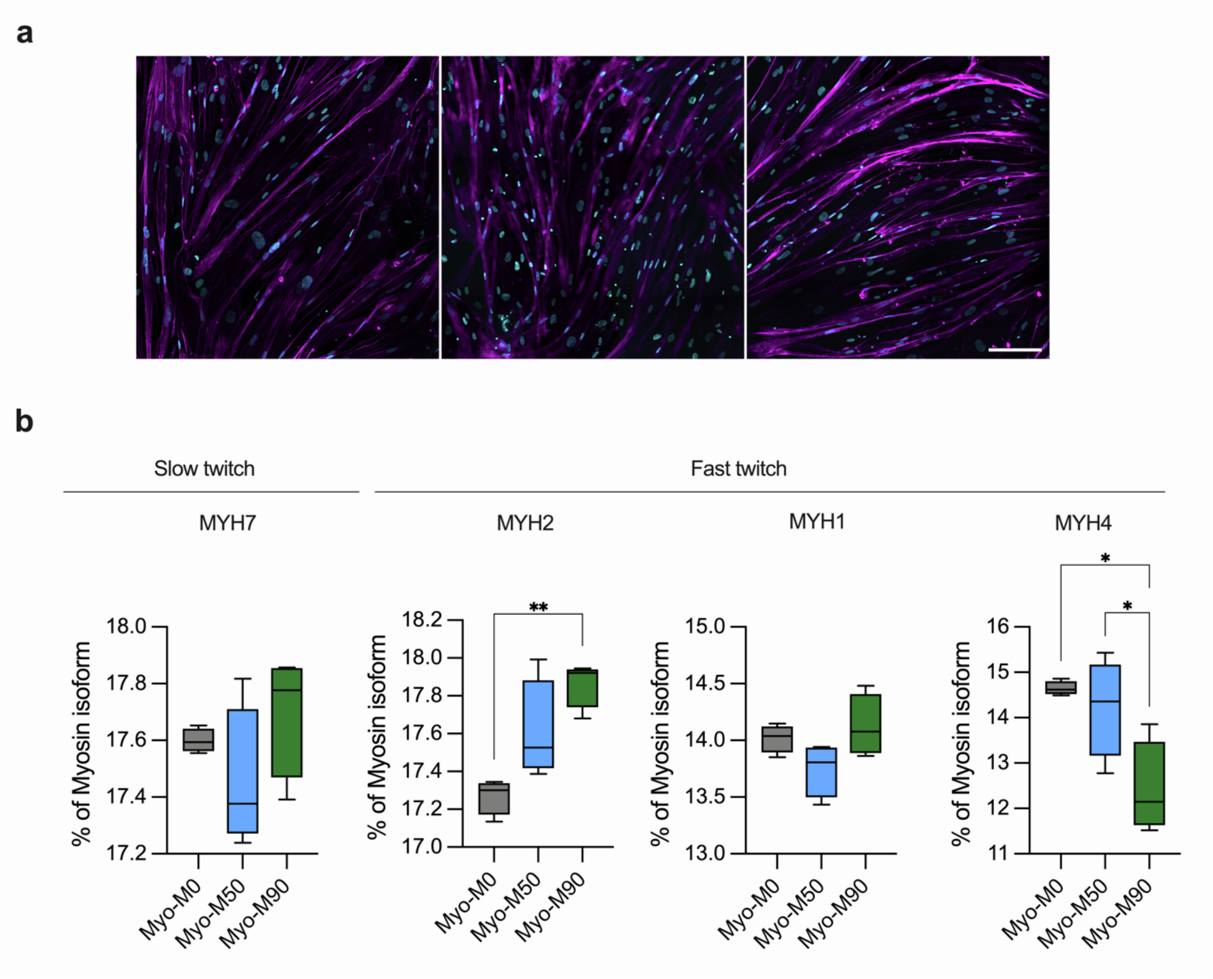

Supplementary Figure 3. Fiber distribution in myofibres expressing the m.3243A>G

(A) Representative images used to quantify nuclei per cell in Figure 3A.

(B) Comparison of the protein abundance obtained from the proteomic analysis. $n = 3$ independent replicates.

Source data are provided as a Source Data file. All data were represented as mean ± SD and data were analysed by one-way ANOVA with Tukey's multiple comparisons test (*p < 0.05, **p < 0.01, ***p < 0.001, ****p < 0.0001).

Fig S4

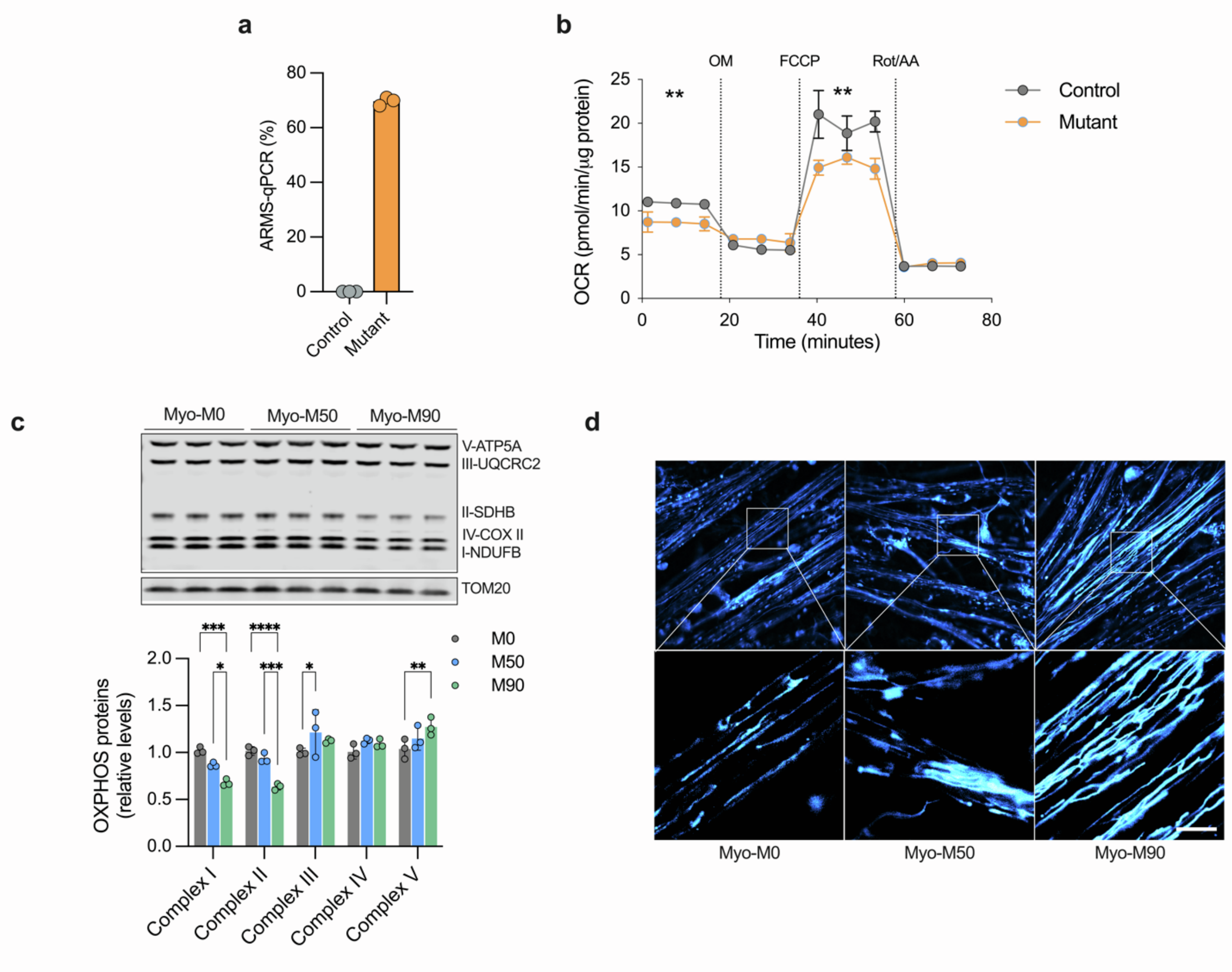

Supplementary Figure 4. Fiber distribution in myofibres expressing the m.3243A>G.

(A) ARMS-qPCR of unrelated control and mutant lines expressing the m.3243A>G mutation. $n = 3$ independent biological replicates.

(B) Cell respiratory capacity measured using the Seahorse XFe96 extracellular flux analyser in myofibres ($n = 3$, 6 culture wells per experiment).

(C) Protein expression of mitochondrial respiratory complexes subunits ($n = 3$ independent biological replicates). TOM20 was used as loading control. Images are representative of at least three independent experiments.

(D) Representative confocal images of NAD(P)H autofluorescence. Scale bar = 100 µM.

Fig S5

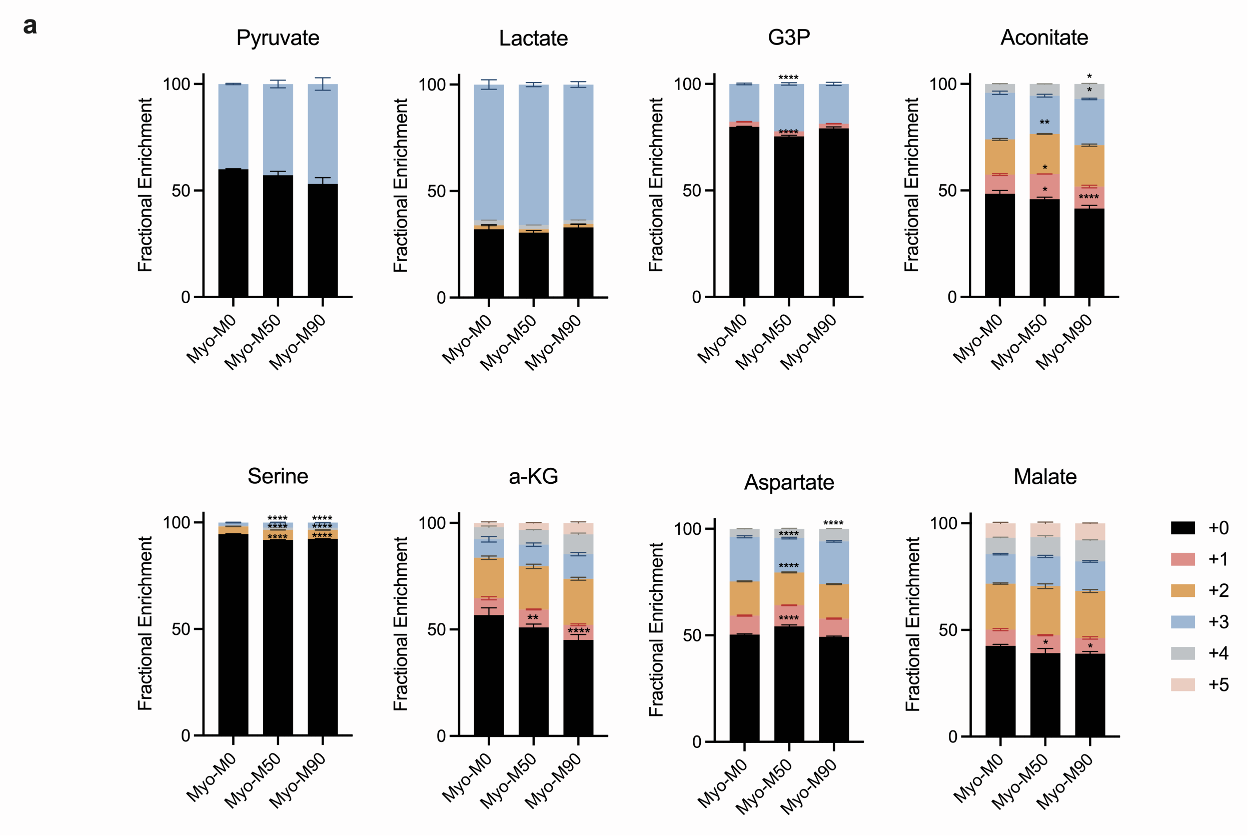

Supplementary Figure 5. Targeted metabolomic analysis showed changes in labelling pattern of metabolites

(A) Fractional enrichment of 13C isotopologues measured by targeted metabolomic (*n* = 3 independent biological replicates).

Source data are provided as a Source Data file. All data were represented as mean ± SD and data were analysed by one-way ANOVA with Tukey's multiple comparisons test (*p < 0.05, **p < 0.01, ***p < 0.001, ****p < 0.0001).

SUPPLEMENTAL METHODS

The mitochondrial oxygen consumption rate

Measurements of oxygen consumption were conducted with the Seahorse Bioscience XFe96 bioanalyzer using the Seahorse XF Cell Mito Stress Test Kit (Cat# 103015-100, Agilent). hiPSC and progenitors were seeded on XF96 cell culture microplates (Cat# 102416-100, Agilent). On the day of the experiment, the culture medium was replaced with Seahorse XF Base medium (Cat# 103334-100, Agilent) supplemented with 1 mM pyruvate (Cat# 11360070, Gibco), 2 mM glutamine (Cat# 25030081, Gibco) and 10 mM glucose (Cat# A2494001, Gibco) and incubated for 30 min at 37 °C in a CO_2-free incubator before loading into the Seahorse Analyser. After measuring basal respiration, the drugs oligomycin (1 µM), FCCP (1 µM, 2 µM), and rotenone/antimycin A (0.5 µM/0.5 µM) were added to each well in sequential order. Data were analysed using the XF Cell Mito Stress Test Report Generator. After the assay, protein was extracted from each well and a BCA assay was performed. The normalisation of the experiments is based on the relative protein obtained.

RT–qPCR

RNA was extracted using the Qiagen RNAeasy Kit (Cat# 74104, Qiangen) following the manufacturer's instructions. RNA concentration was quantified by measuring 260 nm absorbance using a nanodrop spectrophotometer. A complementary DNA (cDNA) library was immediately created from 500 ng of the RNA in a reverse transcription (RT) reaction using the SuperScript II (SSII) Reverse Transcriptase (Cat# 18064014, ThermoFisher). Reverse transcription was conducted in accordance with the Applied Biosystems guidelines. The obtained cDNA was then diluted 1:30 in H_2O in order to maintain an appropriate cycle threshold (Ct) range. Gene expression analysis employed SYBR Green JumpStart Taq ReadyMix (Cat# S4438, Sigma-Aldrich) on the CFX-Connect RT–PCR System, utilising CFX Manager Software version 2.1 (Bio-Rad) as per the manufacturer's instructions. The qPCR data were analysed using the delta-delta Ct method. Primer sequences used are specified as follow:

Target	Forward primer	Reverse primer
OCT4	GACAGGGGGAGGGGAGGAGCTAGG	CTTCCCTCCAACCAGTTGCCCCAAAC
SOX2	GGGAAATGGGAGGGGTGCAAAAGAGG	TTGCGTGAGTGTGGATGGGATTGGTG
NANOG	TGCAAGAACTCTCCAACATCCT	ATTGCTATTCTTCGGCCAGTT
ACTB (housekeeping)	CACCATTGGCAATGAGCGGTTC	AGGTCTTTGCGGATGTCCACGT

Western blot

Myofibres were washed with ice-cold PBS once and lysed using 100 µl RIPA buffer (Cat# R0278, Sigma-Aldrich) with Protease and Phosphatase Inhibitor Cocktail (Cat# 78440, ThermoFisher). Cells were then scraped and stored at -80 C. Protein concentration was quantified using the Pierce BCA Assay Kit (Cat# 23227, ThermoFisher). For immunoblotting, 30 µg of protein samples in NuPAGE 4x LDS Sample Buffer (Cat# NP0007, Invitrogen) and 2% β-mercaptoethanol (Cat# 63689, Sigma-Aldrich) were boiled at 99ºC for 5 min. Proteins were separated on 4-12% NuPAGE Bis-Tris polyacrylamide gels (Cat# NP0335, Invitrogen) and transferred onto PVDF membranes (Cat# IPFL00010, Millipore). Membranes were then incubated in Intercept (TBS) Blocking Buffer (Cat# 927-60001, Li-COR Biosciences) for 1 h at room temperature. After addition of primary antibodies diluted in the blocking buffer with 0.1% Tween-20, membranes were incubated overnight at 4ºC on a shaker. Subsequently, membranes were incubated with appropriate secondary antibodies (Li-COR Biosciences; 1:10000; IRDye® 680RD Goat anti-Mouse IgG, #926-68070; IRDye® 800CW Goat anti-Rabbit IgG, #926-32211) for 1 h at room temperature before signals were developed with the LiCOR Odyssey CLx system.

**Antibodies**	**#Cat**	**Provider**	**Dilution**
alpha Actinin 2 Monoclonal Antibody (EA-53)	MA5-49311	ThermoFisher	1:250
Myosin 4 Monoclonal Antibody (MF20), eBioscience™	14-6503-82	ThermoFisher	1:100
OxPhos Human WB Antibody Cocktail	45-8199	ThermoFisher	1:1000
GPD1 Polyclonal antibody	27943-1-AP	Proteintech	1:1000
GPD1L Polyclonal antibody	17263-1-AP	Proteintech	1:1000

GPD2 Polyclonal antibody	17219-1-AP	Proteintech	1:1000
MDH1 Polyclonal antibody	15904-1-AP	Proteintech	1:1000
Anti-beta Actin antibody	ab8227	abcam	1:1000
Anti-p-S6	4858	Cell Signaling Technology	1:1000
Anti-S6	2217	Cell Signaling Technology	1:1000
Anti-p-Akt	9271	Cell Signaling Technology	1:500
Anti-Akt	9272	Cell Signaling Technology	1:1000

Immunofluorescence

Pluripotent stem cells were seeded at single-cell colonies with ROCK inhibitor (Cat# 1254/10, Bio-techne) for one day. Then, cells were washed, and the media was changed daily until colonies appeared in the culture well. The cells were washed three times with 1X PBS (Cat# 14190144, ThermoFisher) and fixed in 4% paraformaldehyde for 15 min at room temperature and permeabilized with 0.1% Triton X-100 (Cat# 85111, ThermoFisher) for 30 min in PBS. The cells were then washed and incubated with primary antibodies (Myosin Skeletal Muscle antibody, MA1-90701; ACTN2 antibody, 14221-1-AP) in 3% BSA overnight at 4 degrees followed by incubation with Alexa Fluor-conjugated secondary antibodies for 1 h at room temperature. After antibody labelling, the coverslips were mounted on a glass slide using ProLong™ Gold Antifade Mounting (Cat# P36930, ThermoFisher) with DAPI and imaged using the confocal microscope as described above. Image post-processing was performed in ImageJ/FIJI.

Spatial organisation of neighbouring myofibers

Progenitors were differentiated in 35 mm glass-bottom dishes. On the day of the experiment, cells were washed twice with PBS (Cat# 14190144, ThermoFisher) and then fixed with 4% PFA. To observe the directionality of the myofibers in culture, the cells were incubated with α-actinin 2 antibody, followed by Alexa Fluor-conjugated secondary antibody incubation. Images were acquired using Zen Black software

(Carl Zeiss) as described above. Then, images were pseudo-coloured and anal according to Mao et al, 2022.

Lentivirus production

Peredox NADH/NAD^+ sensor (Cat# 163060, Addgene) was transfected into HEK293 cells for lentivirus production. Briefly, the cells were seeded at 70-80% confluency in 10 cm dishes the day before transfection. The constructs (1.2 µg) were co-transfected with vesicular stomatitis virus G (600 ng) and pSPAX2 (800 ng) using Lipofectamine 3000 (Cat# L3000001, ThermoFisher). The lentiviral supernatants were collected 2 days after transfection, and then the cleared supernatant was concentrated with Lenti-X Concentrator (Cat# 631232, Takara) and resuspended in 1 mL of DMEM.

The viral suspension was then added to the pluripotent stem cells as described by Diaz-Cuadros et al. (2023). Blasticidin (1 µg/mL) was used to select cells with the integrated construct. Lentivirus transduction was evaluated using a CLARIOstar microplate reader (excitation/emission = 580/610 nm for mCherry, and excitation/emission = 400/513 nm for Peredox).

Medium pH values

Medium pH values were measured based on the ratiometric property of phenol red. Progenitors were grown in 96-well plates with 150 µl of differentiation media and cultured for 5 days. On day 5, the media was replaced, and samples were collected on day 10. On the day of the experiments, the media from each well was transferred to a new 96-well plate, and the absorbance of phenol red at 443 and 570 nm was immediately measured. The higher the absorbance ratios of 443 to 570 nm, the more acidic the media. A BCA assay was done to ensure the same amount of cells/proteins were obtained per culture well.

Targeted metabolomics

Progenitors were seeded in 60 mm dishes and differentiated for 10 days. Myofibres were then cultured in medium containing 5 mM $^{13}C_6$-glucose (Cat# CLM-1396-5, Cambridge Isotope Laboratories) for an additional 18 h before metabolite isolation. Briefly, cells were washed with PBS (Cat# 14190144, ThermoFisher) three times and resuspended in ice-cold extraction buffer (20% ultrapure water, 50% methanol, 30% acetonitrile) at a ratio of 20 × 10^6 cells per ml. Subsequently, the cells were incubated on methanol and dry ice for 15 min, placed on a shaker for an additional 15 min at 4 °C, and then at –20 °C for 1 h. The cell lysate was centrifuged, and the supernatant was collected and transferred to autosampler

glass vials, which were stored at –80 °C. LC-MS analysis was performed using a Q Exactive Quadrupole-Orbitrap mass spectrometer coupled to a Vanquish UHPLC system (Thermo Fisher Scientific). The liquid chromatography system was fitted with a Sequant ZIC-pHILIC column (150 mm × 2.1 mm) and guard column (20 mm × 2.1 mm) from Merck Millipore (Germany) and temperature maintained at 35°C. The sample (3 µL) was separated at a flow rate of 0.1 mL/min. The mobile phase was composed of 10 mM ammonium bicarbonate and 0.15% ammonium hydroxide in water (solvent A), and acetonitrile (solvent B). A linear gradient was applied by increasing the concentration of A from 20 to 80% within 22 min and then maintained for 7 minutes. The mass spectrometer was operated in full MS and polarity switching mode, in the range of 70-1000m/z and resolution 70000. Major ESI source settings were: spray voltage 3.5 kv, capillary temperature 275°C, sheath gas 35, auxiliary gas 5, AGC target 3e6, and maximum injection time 200 ms. For the targeted analysis, the acquired spectra were analysed using XCalibur Qual Browser and XCalibur Quan Browser software (Thermo Scientific).

NAD(P)H autofluorescence

Progenitors were differentiated in 35 mm glass-bottom dishes. On the day of experiments, cells were washed twice with PBS (Cat# 14190144, ThermoFisher) and then incubated in recording media (DMEM no phenol red). Images were captured on a Zeiss 880 confocal microscope, equipped with a 60x UV-VIS oil immersion objective at 37 °C with excitation at 355 nm. Images were acquired under basal conditions, and after the addition of NaCN to a final concentration of 1 µM and FCCP at a final concentration of 1 µM. A wash with PBS (Cat# 14190144, ThermoFisher) was performed between the drug additions, and fresh media were replenished into the culture wells. The images were analysed with Fiji, and the quantification was done following a previous publication (Chi, Bhosale, & Duchen, 2022).
