# Supplementary material for: Metabolic remodeling in hiPSC-derived myofibers carrying the m.3243A>G mutation
Source: Stem Cell Reports. 2025 Mar 13;20(4):102448. doi: 10.1016/j.stemcr.2025.102448 (PMC12069895; doi:10.1016/j.stemcr.2025.102448)
Supplement: Document S2. Article plus supplemental information [file mmc2.pdf]

Stem Cell Reports
Article
OPEN ACCESS

Metabolic remodeling in hiPSC-derived myofibers carrying the m.3243A>G mutation

Gabriel E. Valdebenito,[1,2,*] Anitta R. Chacko,[1,2] Chih-Yao Chung,[1,2] Preethi Sheshadri,[1,2] Haoyu Chi,[1,2] Benjamin O'Callaghan,[3] Monika J. Madej,[3,4] Henry Houlden,[3] Hannah Rouse,[5] Valle Morales,[5] Katiuscia Bianchi,[5] Francesco Saverio Tedesco,[1,6,7] Robert D.S. Pitceathly,[3,8] and Michael R. Duchen[1,2,9,*]
[1]Department of Cell and Developmental Biology, UCL, Gower Street, London WC1E 6BT, UK
[2]Consortium for Mitochondrial Research, UCL, Gower Street, London WC1E 6BT, UK
[3]Department of Neuromuscular Diseases, UCL Queen Square Institute of Neurology, London WC1N 3BG, UK
[4]Ryvu Therapeutics S.A., Krakow, Poland
[5]Bart's Cancer Institute, Faculty of Medicine and Dentistry, Queen Mary University of London, London, UK
[6]Stem Cells and Neuromuscular Regeneration Laboratory, The Francis Crick Institute, 1 Midland Road, London NW1 1AT, UK
[7]Dubowitz Neuromuscular Centre, UCL Great Ormond Street Institute of Child Health & Great Ormond Street Hospital for Children, London, UK
[8]NHS Highly Specialised Service for Rare Mitochondrial Disorders, Queen Square Centre for Neuromuscular Diseases, The National Hospital for Neurology and Neurosurgery, London WC1N 3BG, UK
[9]Lead contact
*Correspondence: gabriel.valdebenito.19@ucl.ac.uk (G.E.V.), m.duchen@ucl.ac.uk (M.R.D.)
https://doi.org/10.1016/j.stemcr.2025.102448

SUMMARY

Mutations in mitochondrial DNA cause severe multisystem disease frequently associated with muscle weakness. The m.3243A>G mutation is the major cause of mitochondrial encephalomyopathy lactic acidosis and stroke-like episodes (MELAS). Experimental models that recapitulate the disease phenotype *in vitro* for disease modeling or drug screening are very limited. We have therefore generated hiPSC-derived muscle fibers with variable heteroplasmic mtDNA mutation load without significantly affecting muscle differentiation potential. The cells exhibit physiological characteristics of muscle fibers and show a well-organized myofibrillar structure. In cells carrying the m.3243A>G mutation, the mitochondrial membrane potential and oxygen consumption were reduced in relation to the mutant load. We have shown through proteomic, phosphoproteomic, and metabolomic analyses that the m.3243A>G mutation variably affects the cell phenotype in relation to the mutant load. This variation is reflected by an increase in the NADH/NAD$^+$ ratio, which in turn influences key nutrient-sensing pathways in the myofibers. This model enables a detailed study of the impact of the mutation on cellular bioenergetics and on muscle physiology with the potential to provide a platform for drug screening.

INTRODUCTION

Mitochondrial myopathies are mitochondrial diseases usually caused by mutations of nuclear or mitochondrial-encoded proteins and characterized by muscle weakness (Ng et al., 2019). The m.3243A>G DNA mutation typically causes a disease known as mitochondrial encephalomyopathy lactic acidosis and stroke-like episodes (MELAS) (Mancuso et al., 2014). The frequency of carriers ranges from 140 to 250 per 100,000 people, whereas the disease itself is far less common, with its prevalence estimated to be 40 to 70 times lower. As a result, many carriers remain clinically asymptomatic or present with mild disease that is not identified as mitochondrial-related (Pickett et al., 2018). Muscle weakness is a major feature and can be profoundly disabling; however, the phenotype observed in m.3243A>G patients is highly intricate, displaying a wide range of manifestations and severities (Shen and Du, 2021; Tranah et al., 2018). The biochemical and physiological consequences of this specific mutation in terminally differentiated tissues remain poorly understood. Cells carrying the m.3243A>G mutation exhibit heteroplasmy, defined as the presence of both wild-type and mutant mitochondrial DNA (mtDNA). Broadly speaking, disease severity correlates with mutant load, although the relationship between genotype and phenotype remains largely unclear. A major hurdle in understanding the pathophysiology of the disease and in finding treatments is the lack of good experimental systems for disease modeling or for drug screening (Ryytty and Hämäläinen, 2023). Currently, there are limited tools available for generating models of pathogenic mtDNA mutations. Gene editing has not yet evolved to the point we can engineer the mitochondrial genome to produce animal models with specific mtDNA mutations (Silva-Pinheiro and Minczuk, 2022).

We have therefore generated human-induced pluripotent stem cells (hiPSCs) from patient-derived fibroblasts carrying the m.3243A>G mutation, and in the present paper, we describe the generation of viable muscle fibers from the hiPSCs by recapitulating key signaling events during myogenesis (Al Tanoury et al., 2021; Chal and Pourquié, 2017).

© 2025 The Authors.
Published by Elsevier Inc. on behalf of International Society for Stem Cell Research.
This is an open access article under the CC BY license (http://creativecommons.org/licenses/by/4.0/).

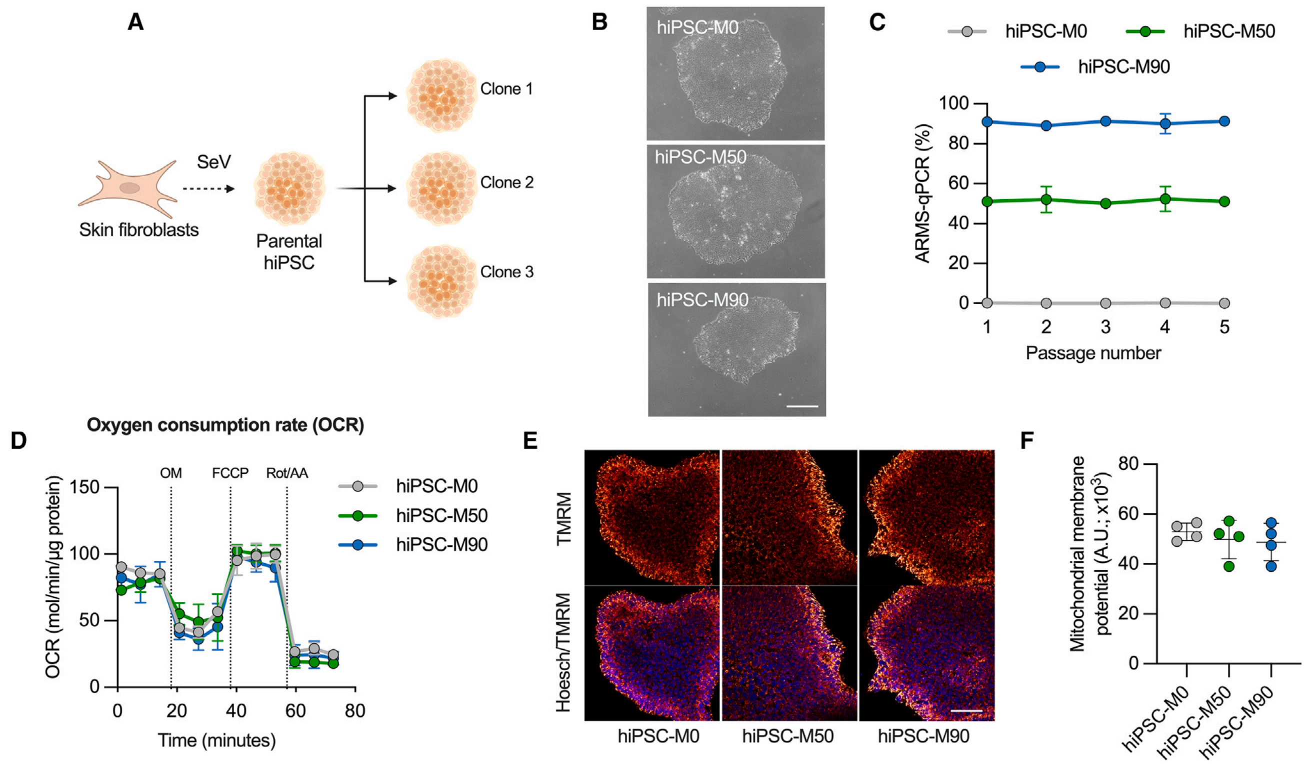

Figure 1. The m.3243A>G mutation remains stable in hiPSCs and does not affect pluripotency or mitochondrial function
(A) Schematic showing experimental approach to select hiPSC colonies carrying different levels of the m.3243A>G mutation. Created with BioRender.com.
(B) Bright-field micrographs of hiPSC colonies in feeder-free conditions. Scale bar, 100 μm.
(C) Mutant load quantification through ARMS-qPCR in hiPSC clones. $n = 3$ independent biological samples.
(D) Cell respiratory capacity measured using the Seahorse XFe96 extracellular flux analyzer in hiPSC colonies. $n = 3$ independent biological samples, 5 culture wells per cell line.
(E) Confocal images of hiPSC loaded with 25 nM tetramethylrhodamine methyl ester (TMRM) and 1μg/mL Hoechst 33342. Scale bar, 150 μM.
(F) Averaged quantification mutant load of hiPSC colonies loaded with TMRM ($n = 4$ independent biological samples). Source data are provided as a Source Data file.
All data were represented as mean ± SD, and data were analyzed by one-way ANOVA with Tukey's multiple comparisons test (*$p < 0.05$, **$p < 0.01$, ***$p < 0.001$, ****$p < 0.0001$).

RESULTS

Implementing an *in vitro* model to study the m.3243A>G mutation

One of the key features of many mtDNA diseases is the expression of heteroplasmy. We took advantage of the random segregation of mtDNA and manually selected clones, which were then expanded and maintained as stable hiPSC lines expressing variable burdens of mutant mtDNA (Figures 1A and 1B). This has the added advantage of enabling the generation of isogenic stem cell lines—a significant advantage in investigating the disease phenotype. Three different cell lines were established, bearing 50% (hiPSC-M50), 90% (hiPSC-M90), and undetectable levels (hiPSC-M0) of the m.3243A>G mutation (Figure 1C). These cell lines exhibited no differences in terms of pluripotency, as all colonies were positive for nuclear markers such as SOX2, NANOG, OCT4, and the surface marker SSEA4 (Figures S1A and S1B). Measurements of gene expression levels through qPCR confirmed the expression of these markers, in contrast to an unrelated human fibroblast line (Figure S1C). The colonies did not exhibit significant differences in terms of oxygen consumption (Figure 1D), measured using the "Seahorse" assay, or mitochondrial membrane potential (Δψm), measured using the equilibration of tetramethylrhodamine methyl ester (TMRM, Figures 1E, 1F, and S1D).

hiPSC bearing the m.3243A>G can successfully differentiate into myofibers

To establish a muscle model that accurately reproduces key characteristics of muscle myopathy in carriers of the m.3243A>G mutation, we employed a differentiation protocol designed to recapitulate crucial signaling events in muscle development. The differentiation strategy involved a combination of fibroblast growth factor 2 (FGF2) and the Wnt agonist CHIR99021 to drive cell commitment toward the mesodermal lineage, while simultaneously inhibiting bone morphogenetic protein signaling with LDN193189 to restrict mesodermal fates to the presomitic mesoderm (Diaz-Cuadros et al., 2020, 2023) (Figure 2A). The induction of all three cell lines was initially assessed at day 7 of differentiation, during which clusters of cells (myocenters) were present in the preparation, surrounded by muscle progenitors (Figure S1E). From day 12 onward, the media were modified by replacing CHIR99021 and LDN193189 with growth factors (hepatocyte growth factor [HGF] and insulin-like growth factor [IGF]) to facilitate the expansion of myogenic progenitors. These progenitors were replated to establish a highly homogeneous cell culture, which was then induced to differentiate into myofibers in a process called secondary myogenesis.

To closely examine this transition and identify potential deviations at the progenitor level caused by the presence of the m.3243A>G mutation, which could potentially interfere with the final differentiation process, we conducted a comparative proteomic analysis between the progenitor cells and myofibers, both with and without the mutation. The score plot of principal component analysis revealed distinct clustering of progenitors and myofibers in separate quadrants of the plot (along PC1), indicating notable differences in these two cell types. Progenitors represent a proliferative cell type, whereas muscle cells are postmitotic and possess a contractile apparatus that is absent in the progenitor population. In the context of the mutant lines, it is noteworthy that the progenitors continued to exhibit close clustering. However, there was a noticeable increase in variance observed between the isogenic control and mutant myofibers (separated along PC2). This heightened variance suggests that the differences in the disease phenotype are significantly more pronounced in postmitotic cells compared to the progenitor cells (Figure 2B), a cell type less dependent on oxidative phosphorylation (Sin et al., 2016).

We also employed k-means clustering algorithm to gain insights into the underlying structure of the dataset (Figure 2C). To elucidate the biological significance of these clusters, we conducted Gene Ontology (GO) analysis. As expected, the top cluster enriched in progenitors (cluster B) contained GO terms associated with proliferative cells, such as the cell cycle, chromosome segregation and organization, as well as mitotic nuclear division. Meanwhile, the bottom cluster (cluster A), highly enriched in myofibers, exhibited a significant enrichment in GO terms related to myofibril assembly, striated muscle contraction, actin filament sliding, and associated processes.

Based on previously reported protein expression profiles in myofibers and progenitors, we curated a list of muscle-related proteins to observe their expression across samples. As expected, isoforms of myosin heavy chain were highly abundant in all myofibers compared to the progenitors. Although not all these myosin isoforms are skeletal muscle specific, some are also expressed in smooth muscle. However, MYH3, MYH7, and MYH8 (the top hits) belong to proteins associated strictly with skeletal muscle specification and maturity (Figures 2D and S2A). Junctophilin-1, which contributes to the construction of the skeletal muscle triad by linking the t-tubule to the sarcoplasmic reticulum, was also highly enriched in myofibers when compared to progenitor cells. LDB3, TNNT1, and ACTN2 were also enriched in the differentiated cells, confirming that all components of the muscle machinery expected in a differentiated myofiber were expressed in the preparation. It is important to note that a few of these proteins were more abundant in the mutant myofibers compared to the isogenic control myofibers. However, when considering all three lines together (Myo-M0, Myo-M50, and Myo-M90), their expression levels were higher than those in their respective progenitors.

We also incorporated proteins associated with cell proliferation into this curated list. Since muscle cells are postmitotic, the expression of these proteins is expected to be lower compared to the progenitors, which are proliferating. The signal-induced proliferation-associated 1/3-like proteins, the upregulator of cell proliferation, and the proliferating cell nuclear antigen were all upregulated in the progenitors. One of the common markers of proliferation, MKI67, was also highly enriched in the progenitors and downregulated across all the myofibers (Figures 2D and S2A).

To understand the impact of the m.3243A>G mutation on muscle structure, we stained fixed preparations of myofibers with an antibody against the heavy chain of myosin II, specifically targeting the light meromyosin portion. Through confocal imaging, we observed positive staining of the myofibers in all preparations (Figure 2E, top panel). Furthermore, we labeled the myofibers with an antibody against alpha-actinin 2, a structural protein expressed in both skeletal and cardiac muscles that serves to anchor myofibrillar actin thin filaments and titin to Z-discs. All fibers were positive for alpha-actinin 2 and showed striations (Figure 2E, bottom panel). To observe the ultrastructure of myofibers more closely, cells were

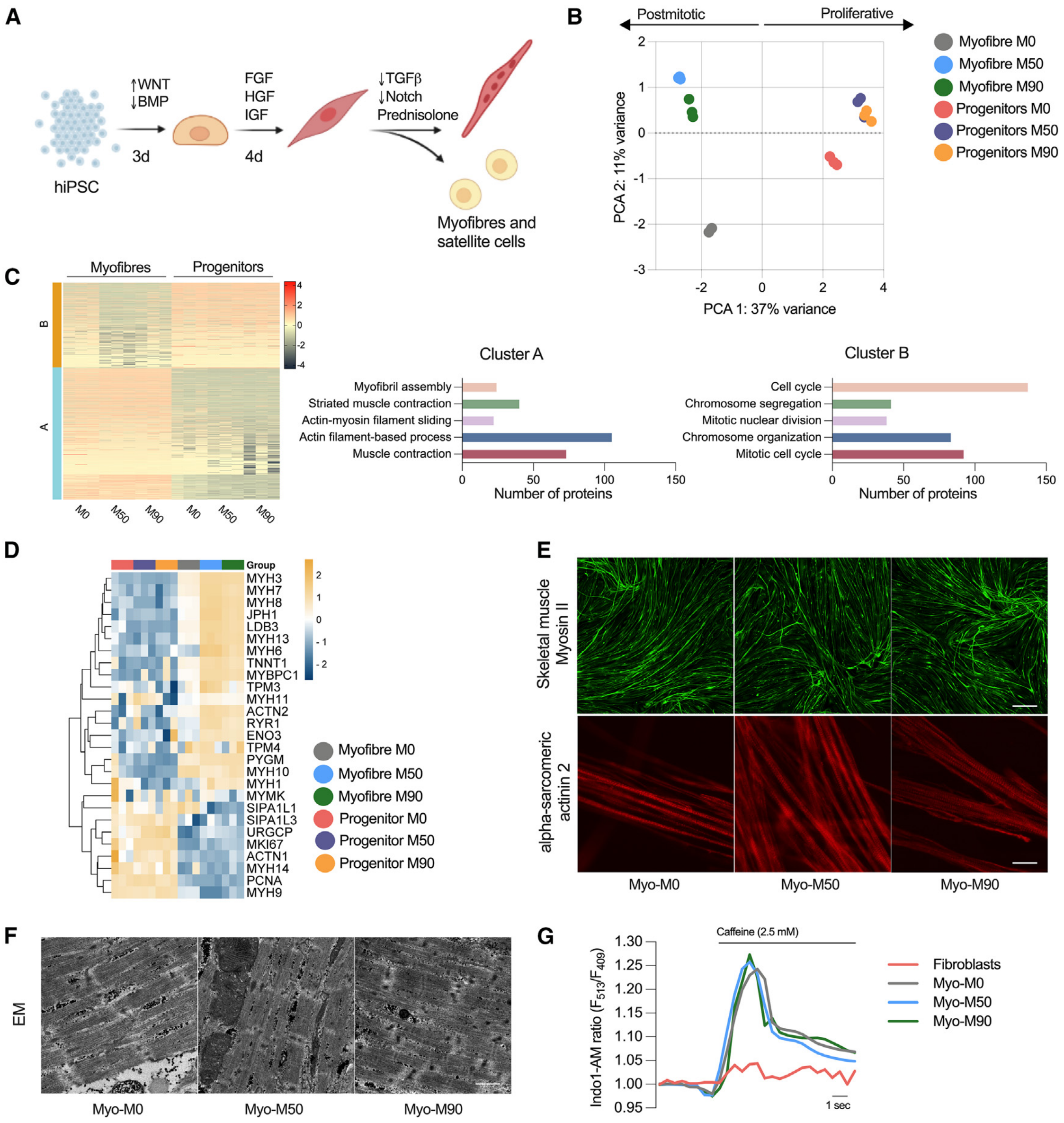

Figure 2. Generation of myofibers derived from hiPSC bearing the m.3243A>G

(A) Protocol used to direct the cells into the mesodermal fate and terminal differentiation of muscle progenitors.

(B) Principal component analysis (PCA) of protein signature showing variance between sample groups (n = 3 replicates per condition).

(C) K-means clustering heatmap of proteins (left, n = 3 replicates) and quantification of the top hits in both clusters.

(D) Heatmap representing proteins associated with muscle maturation in myofibers and progenitors (n = 3 replicates).

(E) Representative confocal images of myofibers stained with antibodies against skeletal muscle myosin (top; scale bar, 500 μM) and α-sarcomeric actinin 2 (bottom; scale bar, 20 μm).

(F) Representative images of electron micrograph sections. At least 10 images were taken per condition. Scale bar, 1 μM.

(G) Representative changes in Indo-1 AM fluorescence intensity after stimulation with 2.5 mM caffeine.

examined by electron microscopy, revealing the presence of sarcomeres along the muscle fibers in all three preparations with no appreciable differences between the different cell lines (Figure 2F). Alpha-actinin staining was pseudo-colored based on local fiber orientation. Assessing the spatial organization of neighboring myofibers revealed aligned bundles, despite the absence of predefined orientation cues (Figure S2B). This observation implies the existence of a self-organizing process during myofiber bundling (Mao et al., 2022).

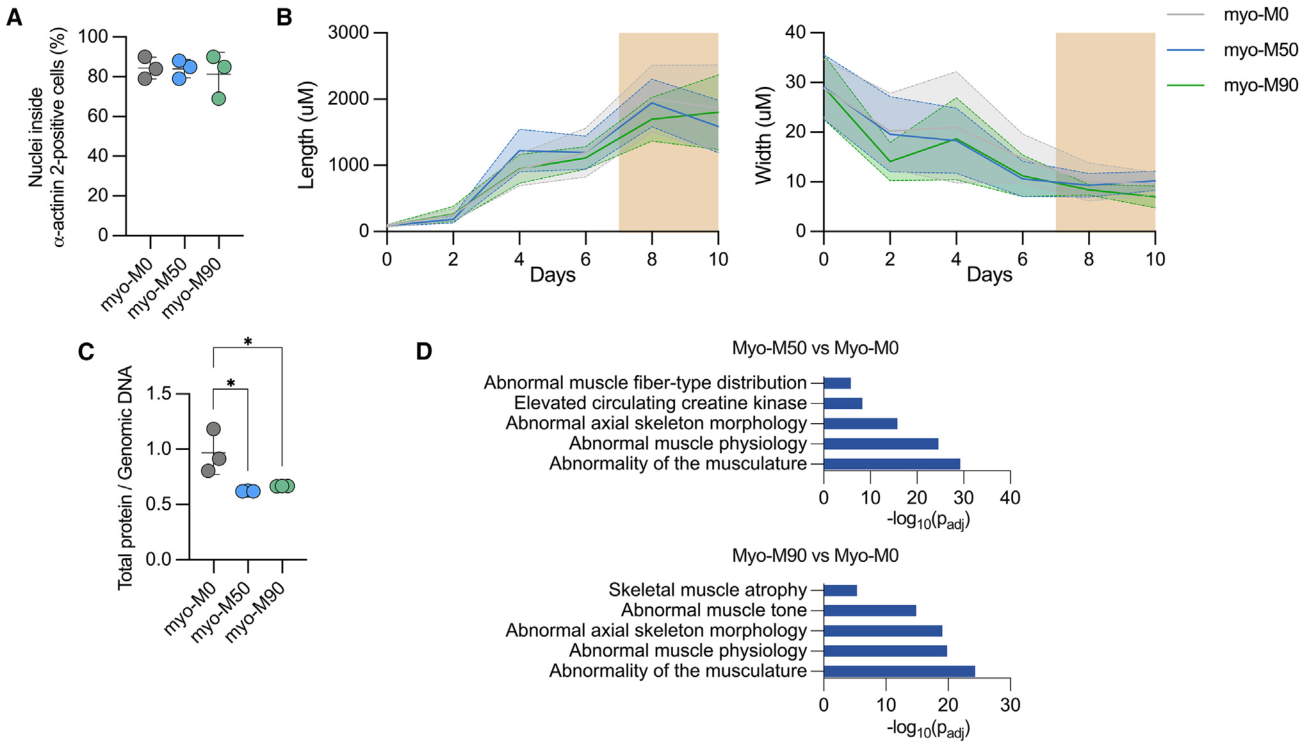

Figure 3. Myogenic efficiency and muscle phenotype in cells carrying the m.3243A>G mutation

(A) Quantification of myogenic differentiation. Nuclei outside and inside α-actinin 2-positive cells are counted and then the ratio of nuclei inside α-actinin 2-positive cells/total nuclei is used to calculate the differentiation efficiency. Results are expressed as the percentage of the total population in the culture ($n = 3$ independent biological replicates).

(B) Length and width of myocytes, myotubes, and myofibers over a period of 10 days of differentiation. Dotted and solid lines show the mean length and width of each day, while shaded area shows standard deviation ($n = 3$ independent biological replicates).

(C) Total cellular protein content relative to genomic DNA in myotubes ($n = 3$ independent biological replicates).

(D) Human Phenotype Ontology of the differentially expressed proteins between Myo-M50 vs. Myo Myo-M0 (top) and Myo-M90 vs. Myo-M0 (bottom). Source data are provided as a Source Data file.

All data were represented as mean ± SD, and data were analyzed by one-way ANOVA with Tukey's multiple comparisons test (*$p < 0.05$, **$p < 0.01$, ***$p < 0.001$, ****$p < 0.0001$).

Muscle contraction is driven by the cytosolic calcium signal, which in turn is shaped by mitochondrial function—both in terms of energy supply and mitochondrial calcium uptake. To test whether the muscle cells were excitable, we first loaded the preparations with the ratiometric calcium indicator Indo-1 AM. All three cell lines showed a response to stimulation with 2.5 mM caffeine, with an increase in cytosolic calcium concentration. There was no difference in the response to caffeine between the isogenic control and mutant myofibers. This response is dependent on the expression of RyR1 Ca^{2+} release channels in the sarcoplasmic reticulum, and no response was measurable in fibroblasts (Figure 2G). These findings show that cell reprogramming is not significantly impaired by the m.3243A>G mutation.

Mitochondrial bioenergetic function is impaired in myofibers carrying the m.3243A>G mutation

Mitochondrial dysfunction is commonly considered a potential hindrance to cellular differentiation as it often impacts development (Qi et al., 2022). To evaluate whether myogenic specification was altered in the *in vitro* model, we quantified the myogenic efficiency by calculating the percentage of nuclei within α-actinin 2-positive cells compared to the total number of nuclei (Figures 3A and S3A). No appreciable differences were observed between the isogenic control and mutant myofibers. We also measured the length and width of myofibers within the preparations. While there were no significant differences in these variables among the three lines during terminal differentiation, mean cell diameter at day 10 was decreased in Myo-M90 cells (Figure 3B). The ratio of total protein

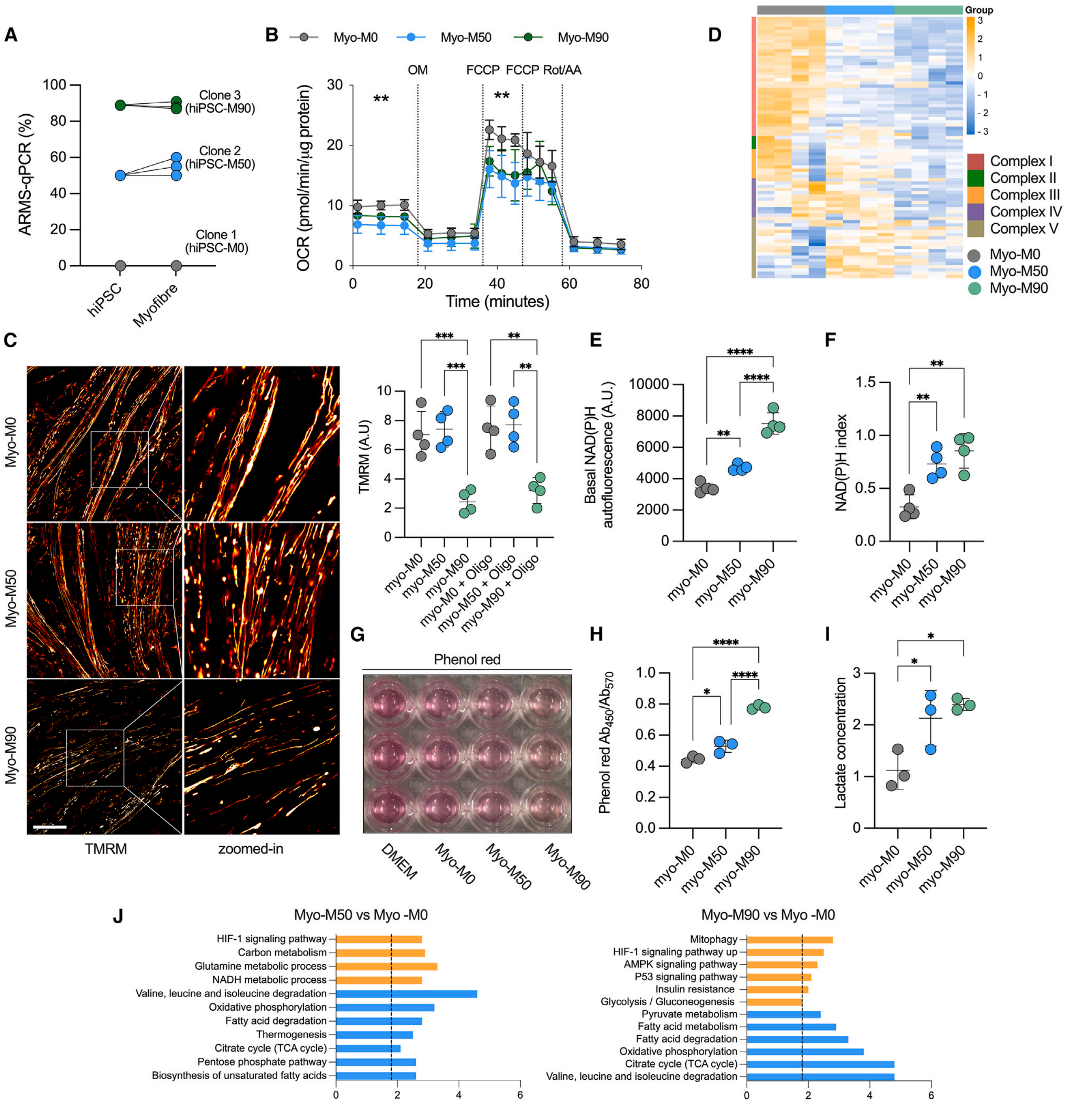

Figure 4. Myofibers expressing the m.3243A>G show mitochondrial dysfunction

(A) Changes in mutation load from hiPSC to fully differentiated myofibers (n = 3 independent biological replicates).

(B) Cell respiratory capacity measured using the Seahorse XFe96 extracellular flux analyzer in myofibers normalized by protein concentration (n = 3, 6 culture wells per experiment).

(C) Confocal images of myofibers loaded with 25 nM tetramethylrhodamine methyl ester (TMRM, left) and quantification of mitochondrial membrane potential (right). n = 4 independent biological replicates. Scale bar, 100 μM.

(D) Proteomic analysis of mitochondrial proteins. n = 4 independent biological replicates.

(E) Levels of basal mitochondrial NAD(P)H measured by NAD(P)H autofluorescence, A.U: arbitrary units. n = 3 independent biological replicates.

(F) Quantification of NAD(P)H redox index. n = 4 independent biological replicates.

(legend continued on next page)

concentration to genomic DNA was decreased in both mutant lines compared to control (Figure 3C). The proteomic analysis also showed enriched terms related to muscle-associated conditions, with "abnormality of the musculature," "abnormal muscle physiology," and "skeletal muscle atrophy" as the top hits (Figure 3D).

To explore the metabolic impact of the m.3243A>G mutation, we first carried out allele refractory mutation system (ARMS)-qPCR to ensure that the mutant load was retained after differentiation. ARMS-qPCR measurements established that mutant load did not vary over time in the different cell lines (Figure 4A). Respiratory rate was measured using the Seahorse XFe96 extracellular flux analyzer. Both basal and maximal uncoupler-induced oxygen consumption rates were significantly reduced in both mutant lines (Figure 4B). To establish that these observations in mitochondrial respiration are common for the m.3243A>G mutation, we differentiated an unrelated hiPSC line carrying a 70% m.3243A>G mutant load detected by ARMS-qPCR (Figure S4A) following the same protocol (Figure 2A) and compared the data against a control hiPSC line derived from a healthy donor. Basal and maximal respiratory rates were also reduced in this mutant cell line (Figure S4B).

Meanwhile, the $\Delta\psi m$ was significantly decreased only in the line carrying the highest mutant load (M90; Figure 4C). To ask whether the $\Delta\psi m$ in Myo-M50 is conserved by the reversal of the F_1F_o-ATP synthase, we treated the cells with oligomycin. In cells in which the ATP synthase works "in reverse" (i.e., as a proton-translocating ATPase), oligomycin causes a collapse of the membrane potential. In the Myo-M50 cells, oligomycin did not alter the $\Delta\psi m$, showing that the potential is not maintained by the reversal of the ATP synthase (McKenzie and Duchen, 2016; Valdebenito et al., 2023). Expression levels of subunits of complex I and II (NDUFB and SDHB) of the electron transport chain (ETC) were decreased in response to the m.3243A>G mutation when measuring representative proteins of the ETC (Figure S4C). To observe global changes in mitochondrial protein expression in Myo-M50 and Myo-M90, we compared the mitochondrial proteome of the ETC components. Notably, we observed that the expression of most of the proteins in complex I was significantly reduced in both mutant lines. This observation was even more pronounced in Myo-M90, where complexes II, III, and IV were also downregulated. Even though complex II is entirely encoded by the nuclear genome, we have noted before in fibroblasts carrying the m.3243A>G mutation that complex II expression was reduced (Chung et al., 2021). Moreover, complexes IV and V were upregulated in Myo-M50 when compared to both the isogenic control and Myo-M90 (Figure 4D).

Complex I is the component of the ETC that oxidizes NADH. Given a decrease in the expression of complex I subunits, we took advantage of the intrinsic fluorescence of NADH to measure the ability of complex I to oxidize this molecule. Under confocal UV excitation, we quantified the basal mitochondrial NADH autofluorescence (Figures 4E and S4D). As expected, both mutant lines showed increased NADH autofluorescence, with a more prominent increase in Myo-M90, consistent with the elevated mutant load and more severely impaired function (Figure 4E). In order to measure changes in the redox state, we treated the cells with NaCN and FCCP to obtain the maximal (fully reduced) and minimal (fully oxidized) autofluorescence signals, respectively. The autofluorescence signal normalized between these values confirmed a more reduced state of the NADH/NAD^+ pool in both mutant lines compared to the isogenic control (Figure 4F).

We observed that the color of phenol red, a pH indicator in the growth media, was more yellow in both cultures of mutant lines compared to the control (Figures 4G and 4H), suggesting acidification consistent with an elevated lactate concentration. This was confirmed when measured using the CuBiAn HT-270 (Figure 4I), recapitulating the lactic acidosis seen in patients with MELAS. GO analysis also suggested the downregulation of tricarboxylic acid (TCA) cycle and oxidative phosphorylation in Myo-M50 and increased glycolysis in Myo-M90, pointing to the reprogramming of these metabolic pathways as a consequence of the m.3243A>G mutation (Figure 4J). Together, these data show that the m.3243A>G mutation alters the bioenergetics of the myofibers, affecting the expression of ETC subunits and promoting increased glycolysis.

Rewiring of nutrient signaling pathways is accompanied by increased NADH/NAD^+ ratio and compensatory changes in NADH shuttles

In the cytosol, the increased rate of glycolysis and NADH generation leads to the conversion of pyruvate into lactate

(G) Representative image of culture wells showing a change in the media color.
(H) Absorbance ratio of phenol red. *n* = 3 independent biological replicates.
(I) Fold changes in lactate concentration measured by CuBiAn. *n* = 3 independent biological replicates.
(J) Top upregulated (orange) and downregulated (blue) Kyoto Encyclopedia of Genes and Genomes (KEGG) pathways from the proteomic dataset. Source data are provided as a Source Data file.
All data were represented as mean ± SD, and data were analyzed by one-way ANOVA with Tukey's multiple comparisons test (**p* < 0.05, ***p* < 0.01, ****p* < 0.001, *****p* < 0.0001).

via lactate dehydrogenase, which restores NAD^+ levels and increases lactate concentration. We quantified the lactate-to-pyruvate ratio reflecting the cytosolic NADH/NAD^+ ratio, which was increased in the mutant lines, with a consistent upregulation in the line carrying the higher mutant load (Figure 5A). We corroborated this observation by expressing the genetically encoded cytosolic NADH/NAD^+ sensor, Peredox, which further confirmed these findings (Figure 5B). Targeted metabolomic analysis also showed changes in the labeled pattern of some metabolites, especially α-ketoglutarate and glycerol-3-phosphate (G3P), which are involved in regulating redox states in the mitochondria and cytosol, respectively (Figure S5).

As cytosolic NADH levels are also influenced by activity of the NADH shuttles, we measured the expression of proteins involved in the G3P and malate-aspartate shuttles (Figure 5C). These shuttles include enzymes such as cytosolic GPD1 (glycerol-3-phosphate dehydrogenase 1), GPD1L (GPD1-like protein), and mitochondrial GPD2 (glycerol-3-phosphate dehydrogenase 2), which play a role in the oxidation of NADH produced during glycolysis. GPD1 utilizes NADH generated by glyceraldehyde 3-phosphate dehydrogenase to convert dihydroxyacetone phosphate (DHAP) into glycerol 3-phosphate. GPD2 then oxidizes G3P back to DHAP while simultaneously reducing flavin adenine dinucleotide and facilitating electron flow through the ETC. The expression of cytosolic GPD1 and its isoform GPD1L increased in relation to the mutant load (Figures 5D and 5E). Conversely, the mitochondrial component of the shuttle exhibited an inverted trend (Figure 5F), decreasing with increased mutant load. Additionally, the ratio between GPD2 and GPD1 revealed a discrepancy in the expression of these enzymes between the mitochondrial and cytosolic compartments (Figure 5G). We also quantified the expression of MDH1, the main component of the malate-aspartate shuttle. MDH1 was increased in the mutant lines, possibly as a compensatory mechanism in response to the increased NADH levels in the cytosol (Figure 5H).

Metabolic rewiring activates the PI3K/AKT/mTORC1 axis in myofibers carrying a high mutant load

We previously reported that in fibroblasts and muscle biopsies from patients carrying the m.3243A>G mutation, the phosphatidylinositol 3-kinase (PI3K)/Akt/mTORC1 pathway was constitutively activated (Chung et al., 2021), apparently serving to sustain the m.3243A>G mutant load (Chung et al., 2024). We therefore assayed the phosphorylation of S6, (a downstream target of mTOR) and AKT (Figure 5I). Consistent with our previous observations, we found that this signaling pathway was significantly upregulated especially in the cell line with the higher mutant load, as evidenced by increased phosphorylation of both AKT and S6 (Figure 5I). In the Myo-M50 mutants, despite a slight increase in S6 phosphorylation, we did not observe any significant differences in AKT phosphorylation levels. To gain a deeper insight into pathway activation, we conducted a comparative phosphoproteomic analysis. AKT indirectly activates mTORC1 by inhibiting the tuberous sclerosis complex $^1/_2$ (TSC1/2), a suppressor of mTORC1 activity. Our findings primarily identified increased phosphorylation of TSC2 (P49815) in Myo-M90, supporting the activation of AKT observed in the Myo-M90 on the western blot. Furthermore, the phosphoproteomic analysis indicated increased phosphorylation of S6 (P62753) at serine 235 and 236 (Figure 5J and 5K), indicating mTORC1 activation, revealing that the m.3243A>G impacts the nutrient-sensing pathway of this model in a different magnitude.

DISCUSSION

In this work, we combined clonal expansion of hiPSCs and functional and omics assays to characterize the metabolic phenotype of hiPSC-derived myofibers carrying the m.3243A>G mutation. The m.3243A>G mutation is the most common mtDNA mutation and varies in frequency across different populations. This mutation can result in maternally inherited diabetes and deafness in some cases, as opposed to the syndrome MELAS. Notably, the m.3243A>G mutation exhibits variability in different cells and tissues of the same person in part due to the presence of mitochondrial heteroplasmy, where cells contain different copies of wild-type and mutated mtDNA (Durham et al., 2007) while maintaining a normal genomic DNA. In this study, we have demonstrated that three clonally expanded lines, despite sharing the same nuclear genetic background, exhibit variable mitochondrial deficiencies and compensations. This variability complicates our understanding of the disease phenotype.

The generation of stem cells carrying m.3243A>G mutation has been used to produce neurons (Hämäläinen et al., 2013; Klein Gunnewiek et al., 2020; Yokota et al., 2017), retinal pigment epithelial (Chichagova et al., 2017), cardiomyocytes (Ryytty et al., 2022; Yokota et al., 2017), neuronal organoids (Khong et al., 2020), endothelial cells (Pek et al., 2019), and other relevant cell types (Ryytty and Hämäläinen, 2023). Nevertheless, it has proven difficult to differentiate stem cells into muscle cells carrying mitochondrial mutations. To generate muscle contraction, this tissue demands a high ATP turnover, comprising a diverse range of cellular processes that activate depending on the intensity and duration of the contraction. Because the muscle stores of ATP are small, the muscle needs to derive energy from phosphocreatine and muscle glycogen

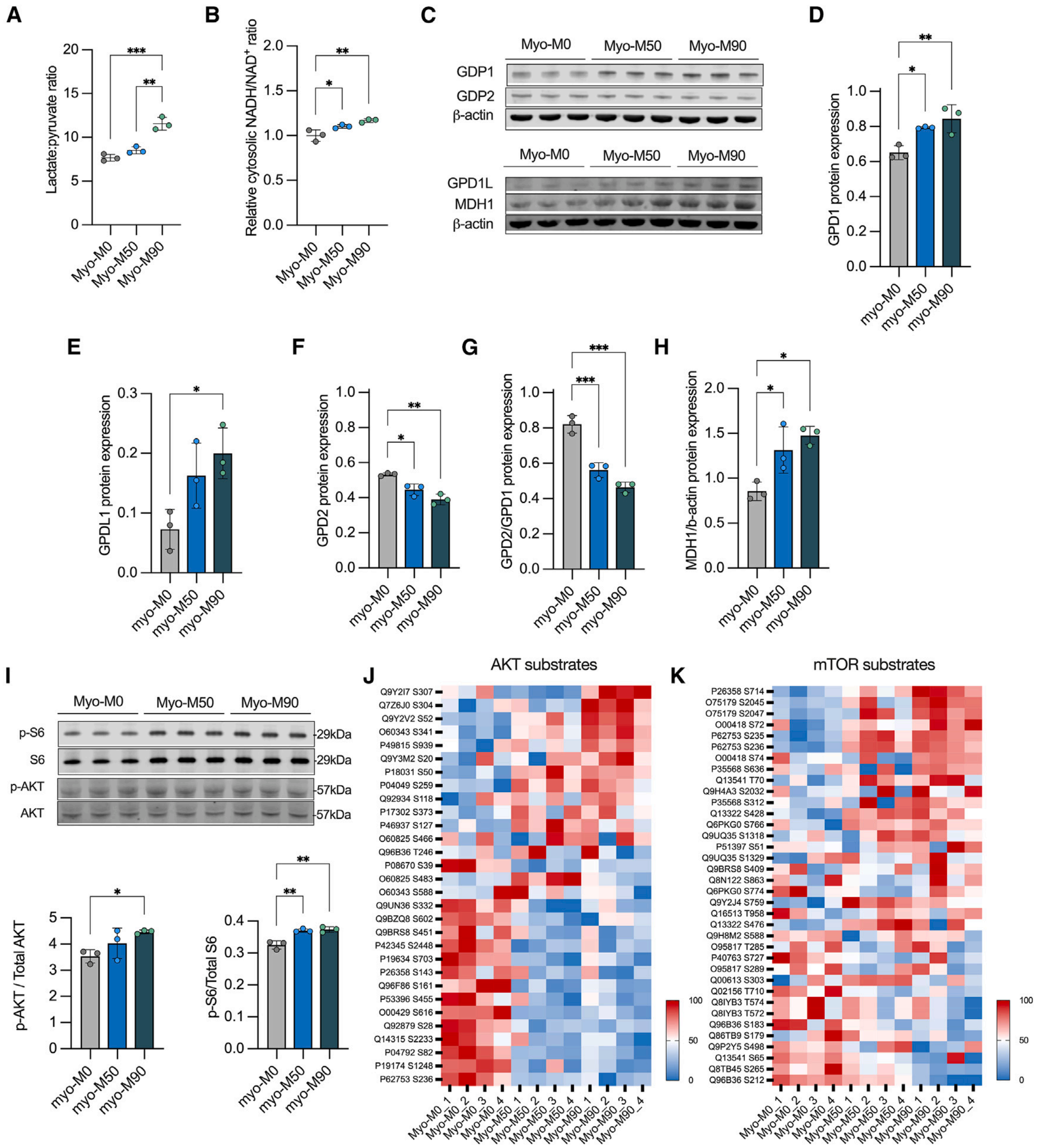

Figure 5. The m.3243A>G rewires cytosolic and mitochondrial metabolism

(A) Lactate to pyruvate ratio obtained from metabolomic analysis. $n = 3$ independent biological replicates.

(B) Relative NADH/NAD^+ ratio obtained from Peredox/mCherry measurements. $n = 3$ independent biological replicates.

(C) Western blot of proteins associated to G3P and MA shuttles.

(D–F and H) Quantification of proteins from (C). $n = 3$ independent biological replicates.

(G) Ratio of protein expression of GPD2/GPD1. $n = 3$ independent biological replicates.

(I) Representative western blot and quantification of phosphorylated S6 (S235/236) and AKT (S473) proteins.

(J and K) AKT and mTOR substrate abundance obtained from phosphoproteomic dataset. $n = 3$ independent biological replicates. Source data are provided as a Source Data file.

All data were represented as mean ± SD, and data were analyzed by one-way ANOVA with Tukey's multiple comparisons test (*$p < 0.05$, **$p < 0.01$, ***$p < 0.001$, ****$p < 0.0001$).

breakdown, enabling substrate-level phosphorylation, and oxidative phosphorylation by utilizing reducing equivalents from carbohydrate and fat metabolism. In a system where mitochondrial ATP is compromised and high glycolytic rates are present, muscle dysfunction may occur, especially when the m.3243A>G mutation affects the majority of mitochondria.

We and others have successfully generated hiPSCs carrying mutations of mitochondrial DNA, and the presence of the m.3243A>G mitochondrial DNA mutations does not interfere extensively with muscle cell reprogramming. This could be explained by the lower dependence observed in pluripotent cells on mitochondrial function. These cells switch to a more glycolytic phenotype, using glucose to sustain rapid cell proliferation and ATP production. Once reprogrammed, we showed that the mutant load remains stable over passage. Others have reported the existence of a potential bottleneck effect during the derivation of cells into the desirable cell type, resulting in a reduction of mutant copies of mtDNA and facilitating normal reprogramming (Chen and Guan, 2023). While our examination did not reveal significant changes in mutant load at these stages, a more detailed investigation into mitochondrial heteroplasmy during differentiation might be valuable. This approach would provide a comprehensive understanding of the dynamics involved in the differentiation process. Notably, despite the absence of major alterations in muscle differentiation, it is essential to highlight the presence of a proteomic signature indicating abnormal fiber distribution and morphology. It is acknowledged that the hiPSC-derived model does not represent a fully developed muscle model; instead, it constitutes a heterogeneous mixture of muscle fiber types. However, this model enables the measurement of key disease features specifically present in muscle tissue and identifies reproducible and robust readouts that could be used for drug screening. This underscores the significance of our findings in elucidating the aspects of disease pathology that might be overlooked in more simplistic models.

While we have reported constitutive activation of the PI3K-AKT-mTOR pathway in fibroblasts, cybrid cells, and muscle biopsies (Chung et al., 2021; Chung et al., 2022), it seems that this phenomenon might be cell type and mutant load-specific as we observed varying degrees of activation in the isogenic lines. Given that this pathway is a nutrient signaling pathway, our line with a mid-range mutant load appears to compensate for mitochondrial dysfunction by increasing TCA activity and ETC proteins. It is important to note that the observations are not linear, as the Myo-M90 line exhibited different behavior. Nevertheless, both cell lines were bioenergetically compromised, as evident in mitochondrial respiration and membrane potential results.

Variation in mtDNA heteroplasmy levels, specifically the 3243A>G mutation, has been described to lead to distinct phenotypic outcomes in patients, with mutant loads of 10%–30% causing diabetes and occasional autism, 50%–90% resulting in encephalomyopathies, and 90%–100% leading to perinatal lethality. This has been attributed to transitions in cellular phenotype and gene expression, revealed through analyses of cybrids with increasing mutant mtDNA levels (Picard et al., 2014).

Overall, we have demonstrated the possibility of generating a muscle model derived from hiPSC expressing variable levels of the m.3243A>G mutation. This model showed that, regardless of the high levels of mutant DNA, the cells express a muscle phenotype characterized by contractility in response to chemical and electrical stimulation, given the formation of fully assembled sarcomeres. With this, we were able to explore mitochondrial dysfunction, where we observed changes in key mitochondrial variables, such as mitochondrial membrane potential and mitochondrial respiration. As expected, we obtained a higher mitochondrial NADH accumulation since the ETC is not able to fully oxidize this molecule, regulating TCA cycle flux. We also observed a major cytosolic NADH content in the mutant lines, suggesting an increased activity of NADH shuttles to replenish NAD^+ content. Finally, these parameters affect nutrient-sensing signaling pathways to a different degree, as reported in other cell models, which is corroborated with phosphoproteomics assays.

METHODS

A detailed description of the procedures is provided in supplemental methods.

Reprogramming of human dermal fibroblasts to hiPSCs

Fibroblasts were obtained from the MRC Centre for Neuromuscular Diseases Biobank. Cells were reprogrammed into hiPSCs through non-integrative delivery of hOCT4, hSOX2, hKLF4, and hc-MYC using the CytoTune-iPS 2.0 Sendai Reprogramming Kit (Cat# A16517, Thermo Fisher Scientific). At all stages, cells were maintained in a humidified incubator at 37°C, 95% air/5% CO_2 gas mixture.

One day prior to reprogramming induction (d-1): 4×10^5 fibroblasts at a passage number <10 were seeded into a single well of a 6-well plate and cultured for 24 h in MEF media: Dulbecco's modified Eagle's medium:nutrient mixture F12 supplemented with GlutaMAX (DMEM/F12; Gibco) and 10% v/v fetal bovine serum (Gibco). The following day, a transduction mixture was prepared in 2 mL of MEF medium consisting of CytoTune 2.0 Sendai vectors hKOS, hc-Myc, and hKLF4 at a multiplicity of infection ratio of

1:1:0.6. 24 h later, the transduction mixture was removed, and cells were washed once with DPBS before adding fresh MEF medium. Cells were then cultured for 5 days without medium change. Then, transduced fibroblasts were split with 0.05% w/v trypsin-EDTA (Gibco) and seeded in MEF medium onto a 10 cm dish prepared with irradiated CF1 MEFs (GlobalStem). 24 h after seeding of the transduced fibroblasts onto MEFs (day 7), medium was changed to a knockout serum replacement (KSR) feeder medium consisting of DMEM/F12 supplemented with 10% v/v KSR (Gibco), 1x minimum essential medium non-essential amino acids (Gibco), 55 mM β-mercaptoethanol (Gibco), and 4 ng/mL human FGF2 (hFGF-2; R&D Systems). KSR feeder medium was changed daily until hiPSC colonies with a diameter of ∼2–4 mm were visible (3–4 weeks).

Isolation of clonal hiPSC lines

Colonies were visualized using an inverted microscope and mechanically divided into at least four equally sized clumps using a 10 μL pipette tip. The clumps were then transferred to a single well of a 24-well plate prepared with feeders. KSR feeder medium was additionally supplemented with 10 μM Rho-associated, coiled-coil-containing protein kinase inhibitor (ROCKi) Y-27632 (Sigma) to promote the survival of dissociated cells. Medium was replaced the following day without the addition of ROCK inhibitor and daily thereafter until ∼60% confluency. hiPSCs reprogrammed under feeder-dependent conditions were then transferred to standard feeder-free conditions.

Maintenance of hiPSCs

hiPSCs were cultured in mTeSR plus (Cat# 100–0276, STEMCELL Technologies) on Matrigel-coated plates (Cat# 354230, Corning Life Sciences) until they reached 70% confluency. Subsequently, cells were split using ReleSR (Cat# 100-0483, STEMCELL Technologies) in a 1:6 ratio. To prevent cell death, a 10 μM ROCK inhibitor (Cat# 1254/10, Bio-techne) was applied for 1 day. Daily media changes were performed, and the cultures were cleaned with a pipette tip under a microscope (EVOS XL Core Imaging System) to eliminate spontaneously differentiated cells that arose randomly around the edge of the colonies.

Skeletal myogenic differentiation of hiPSCs

The direct reprogramming process was conducted based on an adaptation of Chal et al. Initially, hiPSC cultures were maintained in mTeSR plus (Cat# 100-0276, STEMCELL Technologies) on Matrigel-coated surfaces (Cat# 354230, Corning Life Sciences) until reaching 100% confluence before initiating myogenic differentiation. At this confluency, hiPSC cells were replated into an isolated cell suspension with 10 μM ROCK inhibitor (Cat# 1254/10, Bio-techne) at a very low density (no more than 12 cells per colony) and incubated overnight. The following day, the media was changed to remove the ROCK inhibitor and was maintained for 6 h in mTeSR plus (Cat# 100-0276, STEMCELL Technologies). The media was then replaced with DMEM/F12 (Cat# 11320033, Thermo Fisher Scientific) supplemented with Insulin-Selenium-Transferrin (Cat# 41400045, Thermo Fisher Scientific), 3 μM CHIR99021 (Cat# 4423/10, Bio-techne), and 500 nM LDN193189 (Cat# 04-0074-10, Generon). Media changes were performed every 2 days. On day 5, the media was supplemented with 20 ng/mL hFGF-2 (Cat# 450-33, PeproTech) for 3 additional days. Starting from day 6, the media was switched to DMEM/F12 supplemented with 15% KSR (Cat# 10828028, Thermo Fisher Scientific), 10 ng/mL HGF (Cat# 315-23, PeproTech), 2 ng/mL IGF-1 (Cat# 250-19, PeproTech), 20 ng/mL FGF2 (Cat# 450-33, PeproTech), and 500 nM LDN193189 (Cat# 04-0074-10, Generon). From day 8, DMEM/F12 was supplemented with 15% KSR and 2 ng/mL IGF-1 until day 12. On day 12, the media was supplemented with 10 ng/mL HGF until day 22. Subsequently, cells were re-plated in skeletal muscle growth medium-2 (Cat# CC-3245, Lonza) for expansion. The medium was refreshed every 2 days until reaching 80% confluence. Myogenic progenitors were harvested and cryopreserved for downstream applications or replated for experimentation. All cultures were maintained in humidified air supplemented with 5% CO_2 at 37°C.

Measurement of mtDNA mutant load

Levels of mtDNA mutation were detected using an ARMS-based qPCR analysis. DNA extractions from cells were performed using the DNeasy Blood & Tissue Kit (Cat# 69506, QIAGEN). The concentrations of DNA samples were quantified using NanoDrop. Samples were diluted to 0.4 ng/μL. ARMS-qPCR primer working solutions (5 μM, 1 μL each; forward [3243A]: CAGGGTTTGTTAAGATG GCAtA; forward [3243G]: CAGGGTTTGTTAAGATGG CAtG; reverse: TGGCCATGGGTATGTTGTTA) and SYBR Green JumpStart Taq Ready Mix (Cat# S4438, Sigma-Aldrich) were combined to create master mixes for mutant and wild-type genes. DNA samples (3 μL) and master mixes (7 μL) were pipetted into a 96-well PCR plate (Cat# MLL9651, Bio-Rad), and PCR amplification was performed using the CFX96 Touch Real-Time PCR Detection System (Bio-Rad). Each sample had three technical replicates. The mutant heteroplasmy level (%) was calculated using a previously described method, as shown in the following equation.

$$Mutant\ load\ \% = \frac{1}{1+(1+2)^{\Delta Ct}} \times 100$$

Mitochondrial membrane potential

Progenitors were seeded in 35 mm fluorodishes and differentiated until day 10. Myofibers were washed twice with the phenol-free recording medium (Cat# A1443001, Gibco) with 10 mM glucose, 1 mM glutamine, and 10 mM HEPES, adjusted to pH 7.4, and then incubated with 25 nM TMRM for 30 min at 37°C. Cells were imaged with an LSM 880 (Carl Zeiss) confocal microscope using Fluar 63x/1.40 oil immersion objective lens at 37°C. TMRM was excited with a 561 nm Argon laser with an output power of 0.2 mW. MBS 488/561 was used as a beam splitter and emitted fluorescence collected at 564–740 nm. Images were acquired using Zen Black software (Carl Zeiss), and fluorescence intensity was quantified using Fiji with the same threshold across all samples.

Mass spectrometry-based bulk proteomics and phosphoproteomics

Myofibers and progenitors were grown in 6-well plates. Culture wells were washed twice with PBS (Cat# 14190144, Thermo Fisher Scientific), and subsequently, RIPA buffer and 1X proteinase and phosphatase inhibitors were added directly to the plate. The cell lysate was collected by scraping the plate and boiled for an additional 10 min followed by microtip probe sonication for 2 min with pulses of 1 s on and 1 s off at 80% amplitude. Protein concentration was estimated by BCA.

Proteomic and phosphoproteomic data analysis

The original data were first $\log_2$ transformed, and then only the proteins with at least 3 values from the 4 replicates were kept. At this point, the missing data were imputed using automatic settings of Perseus. The proteomic dataset was then analyzed using ExpressVis (Liu et al., 2022), and data visualization was done using GraphPrism and SRplot (Tang et al., 2023). The phosphoproteomic dataset was analyzed using Phosphomatics (Leeming et al., 2021), and data visualization was done using GraphPrism and SRplot (Tang et al., 2023). The mass spectrometry proteomics and phosphoproteomic data have been deposited to the ProteomeXchange Consortium via the PRIDE (Perez-Riverol et al., 2022) partner repository with the dataset identifier PXD058785.

Calcium imaging

For imaging of cytoplasmic calcium, cells were washed once with phenol-free DMEM containing HEPES (Cat# 21063029, Gibco), then incubated with 1 μM Indo-1 AM (Cat# I1223, Invitrogen) and 0.02% Pluronic F-127 (Cat# P2443, Sigma-Aldrich) for 30 min at 37°C. Cells were then washed and incubated for further 20 min in phenol-free DMEM at 37°Cs to allow de-esterification of intracellular AM esters. Cells were imaged using a UV-vis Zeiss LSM 880 confocal microscope equipped with a 20× objective. Indo-1 fluorescence was excited at 355 nm, and emission measured simultaneously at 390 and 495 nm for Ca^{2+}-bound Indo-1 and unbound Indo-1, respectively.

Images were analyzed using ImageJ/Fiji. Regions of interest (ROIs) were manually selected for each cell (at least 30 cells per technical replicate), and mean fluorescence intensity was quantified for all ROIs in each channel. Background was subtracted, and ratios between the emission signals of bound/unbound Indo-1 were calculated over time. The resulting ratioed traces representing cytosolic $[Ca^{2+}]c$ levels have been plotted.

Quantification of myogenic differentiation

Quantification of myogenic differentiation was performed as described in a previous publication (Maffioletti et al., 2015). In brief, we quantified the percentage by manually counting nuclei in single images of the different lines shown in Figure S3A. Nuclei inside and outside actinin-2-positive cells were counted, and the ratio of nuclei inside actinin-2-positive cells to total nuclei was used to calculate differentiation efficiency. A total of five images, each containing more than 20 fibers, were quantified per differentiation set.

RESOURCE AVAILABILITY

Lead contact

Further information and requests for resources and reagents should be directed to and will be fulfilled by the lead contact, Michael Duchen (m.duchen@ucl.ac.uk).

Materials availability

This study did not generate new unique reagents.

Data and code availability

The mass spectrometry proteomics and phosphoproteomic data have been deposited to the ProteomeXchange Consortium via the PRIDE (Perez-Riverol et al., 2022) partner repository with the dataset identifier: PXD058785.

ACKNOWLEDGMENTS

We thank Lu Yan and Olivier Pourquié for the muscle differentiation protocol. We thank Riccardo Zenezini and the UCL Mass-Spectrometry Science Technology Platform for the proteomic and phosphoproteomic analysis. We acknowledge the metabolic flux analysis facility of the Barts School of Medicine and Dentistry created with the support of the Barts and the London Charity, grant number MGU0401. We acknowledge Dr. Monika Madej for the generation of the hiPSC clones. F.S.T. acknowledges support of the European Research Council (759108 – HISTOID). R.D.S.P. is funded by The Lily Foundation, Muscular Dystrophy UK (MDUK), and a seedcorn award from the Rosetrees Trust and Stoneygate Foundation. R.D.S.P. is supported by a Medical Research Council (UK) Transition Support award (MR/X02363X/1), a

Medical Research Council (UK) award (MC_PC_21046) to establish a National Mouse Genetics Network Mitochondria Cluster (MitoCluster), and the LifeArc Centre to Treat Mitochondrial Diseases (LAC-TreatMito). R.D.S.P. and H.H. are supported by a Medical Research Council strategic award (MR/S005021/1) to establish an International Centre for Genomic Medicine in Neuromuscular Diseases (ICGNMD). The University College London Hospitals/ University College London Queen Square Institute of Neurology sequencing facility receives a proportion of funding from the Department of Health's National Institute for Health Research Biomedical Research Centre funding scheme. The clinical and diagnostic "Rare Mitochondrial Disorders" Service in London is funded by the UK NHS Highly Specialised Commissioners. Early work in this area was supported by funding from Action Medical Research. A.R.C. is supported by the Medical Research Council, MRC DTP-iCASE programme (MR/RO15759/1). G.E.V. is supported by the National Agency for Research and Development (ANID)/Scholarship Program/DOCTORADO BECAS CHILE/ 2019 – 7220052. We thank bit.bio for their financial support during the development of this work.

AUTHOR CONTRIBUTIONS

G.E.V. and M.R.D. conceived the project and designed and performed the experiments. A.R.C., C.-Y.C., P.S., M.J.M., and B.O. provided resources and performed the experiments. H.R., V.M., and K.B. performed and analyzed metabolomic data. H.H., F.S.T., and R.D.S.P. provided expert input on experimental design and analysis. G.E.V. and M.R.D. wrote the manuscript. All authors reviewed and approved the final version.

DECLARATION OF INTERESTS

The authors declare no competing interests.

SUPPLEMENTAL INFORMATION

Supplemental information can be found online at https://doi.org/10.1016/j.stemcr.2025.102448.

Received: December 18, 2024
Revised: February 11, 2025
Accepted: February 12, 2025
Published: March 13, 2025

REFERENCES

Al Tanoury, Z., Zimmerman, J.F., Rao, J., Sieiro, D., McNamara, H.M., Cherrier, T., Rodríguez-delaRosa, A., Hick-Colin, A., Bousson, F., Fugier-Schmucker, C., et al. (2021). Prednisolone rescues Duchenne muscular dystrophy phenotypes in human pluripotent stem cell-derived skeletal muscle *in vitro*. Proc. Natl. Acad. Sci. USA *118*, e2022960118. https://doi.org/10.1073/pnas.2022960118.

Chal, J., and Pourquié, O. (2017). Making muscle: skeletal myogenesis *in vivo* and *in vitro*. Development *144*, 2104–2122. https://doi.org/10.1242/dev.151035.

Chen, C., and Guan, M.X. (2023). Induced pluripotent stem cells: ex vivo models for human diseases due to mitochondrial DNA mutations. J. Biomed. Sci. *30*, 82. https://doi.org/10.1186/s12929-023-00967-7.

Chichagova, V., Hallam, D., Collin, J., Buskin, A., Saretzki, G., Armstrong, L., Yu-Wai-Man, P., Lako, M., Steel, D.H., and Steel, D.H. (2017). Human iPSC disease modelling reveals functional and structural defects in retinal pigment epithelial cells harbouring the m.3243A > G mitochondrial DNA mutation. Sci. Rep. *7*, 12320. https://doi.org/10.1038/s41598-017-12396-2.

Chung, C.Y., Singh, K., Kotiadis, V.N., Valdebenito, G.E., Ahn, J.H., Topley, E., Tan, J., Andrews, W.D., Bilanges, B., Pitceathly, R.D.S., et al. (2021). Constitutive activation of the PI3K-Akt-mTORC1 pathway sustains the m.3243 A > G mtDNA mutation. Nat. Commun. *12*, 6409. https://doi.org/10.1038/s41467-021-26746-2.

Chung, C.Y., Singh, K., Sheshadri, P., Valdebenito, G.E., Chacko, A.R., Costa Besada, M.A., Liang, X.F., Kabir, L., Pitceathly, R.D.S., Szabadkai, G., et al. (2024). Inhibition of the PI3K-AKT-MTORC1 axis reduces the burden of the m.3243A>G mtDNA mutation by promoting mitophagy and improving mitochondrial function. Autophagy, 1–16. https://doi.org/10.1080/15548627.2024.2437908.

Chung, C.Y., Valdebenito, G.E., Chacko, A.R., and Duchen, M.R. (2022). Rewiring cell signalling pathways in pathogenic mtDNA mutations. Trends Cell Biol. *32*, 391–405. https://doi.org/10.1016/j.tcb.2021.10.005.

Diaz-Cuadros, M., Miettinen, T.P., Skinner, O.S., Sheedy, D., Díaz-García, C.M., Gapon, S., Hubaud, A., Yellen, G., Manalis, S.R., Oldham, W.M., et al. (2023). Metabolic regulation of species-specific developmental rates. Nature *613*, 550–557. https://doi.org/10.1038/s41586-022-05574-4.

Diaz-Cuadros, M., Wagner, D.E., Budjan, C., Hubaud, A., Tarazona, O.A., Donelly, S., Michaut, A., Al Tanoury, Z., Yoshioka-Kobayashi, K., Niino, Y., et al. (2020). In vitro characterization of the human segmentation clock. Nature *580*, 113–118. https://doi.org/10.1038/s41586-019-1885-9.

Durham, S.E., Samuels, D.C., Cree, L.M., and Chinnery, P.F. (2007). Normal levels of wild-type mitochondrial DNA maintain cytochrome c oxidase activity for two pathogenic mitochondrial DNA mutations but not for m.3243A–>G. Am. J. Hum. Genet. *81*, 189–195. https://doi.org/10.1086/518901.

Hämäläinen, R.H., Manninen, T., Koivumäki, H., Kislin, M., Otonkoski, T., and Suomalainen, A. (2013). Tissue- and cell-type-specific manifestations of heteroplasmic mtDNA 3243A>G mutation in human induced pluripotent stem cell-derived disease model. Proc. Natl. Acad. Sci. USA *110*, E3622–E3630. https://doi.org/10.1073/pnas.1311660110.

Khong, Z.J., Soh, B.S., Fan, Y., and Ng, S.Y. (2020). Organoid cultures of MELAS neural cells reveal hyperactive Notch signaling that impacts neurodevelopment. Cell Death Dis. *11*, 182. https://doi.org/10.1038/s41419-020-2383-6.

Klein Gunnewiek, T.M., Van Hugte, E.J.H., Frega, M., Guardia, G.S., Foreman, K., Panneman, D., Mossink, B., Linda, K., Keller, J.M., Schubert, D., et al. (2020). m.3243A > G-Induced Mitochondrial Dysfunction Impairs Human Neuronal Development and Reduces Neuronal Network Activity and Synchronicity. Cell Rep. *31*, 107538. https://doi.org/10.1016/j.celrep.2020.107538.

Leeming, M.G., O'Callaghan, S., Licata, L., Iannuccelli, M., Lo Surdo, P., Micarelli, E., Ang, C.S., Nie, S., Varshney, S., Ameen, S., et al. (2021). Phosphomatics: interactive interrogation of substrate-kinase networks in global phosphoproteomics datasets. Bioinformatics *37*, 1635–1636. https://doi.org/10.1093/bioinformatics/btaa916.

Liu, X., Xu, K., Tao, X., Yin, R., Ren, G., Yu, M., Li, C., Chen, H., Zhao, K., Xiang, S., et al. (2022). ExpressVis: a biologist-oriented interactive web server for exploring multi-omics data. Nucleic Acids Res. *50*. W312–w321. https://doi.org/10.1093/nar/gkac399.

Maffioletti, S.M., Gerli, M.F.M., Ragazzi, M., Dastidar, S., Benedetti, S., Loperfido, M., VandenDriessche, T., Chuah, M.K., Tedesco, F.S., and Tedesco, F.S. (2015). Efficient derivation and inducible differentiation of expandable skeletal myogenic cells from human ES and patient-specific iPS cells. Nat. Protoc. *10*, 941–958. https://doi.org/10.1038/nprot.2015.057.

Mancuso, M., Orsucci, D., Angelini, C., Bertini, E., Carelli, V., Comi, G.P., Donati, A., Minetti, C., Moggio, M., Mongini, T., et al. (2014). The m.3243A>G mitochondrial DNA mutation and related phenotypes. A matter of gender? J. Neurol. *261*, 504–510. https://doi.org/10.1007/s00415-013-7225-3.

Mao, Q., Acharya, A., Rodríguez-delaRosa, A., Marchiano, F., Dehapiot, B., Al Tanoury, Z., and Schnorrer, F. (2022). Tension-driven multi-scale self-organisation in human iPSC-derived muscle fibers. Elife *11*, e76649. https://doi.org/10.7554/eLife.76649.

McKenzie, M., and Duchen, M.R. (2016). Impaired Cellular Bioenergetics Causes Mitochondrial Calcium Handling Defects in MT-ND5 Mutant Cybrids. PLoS One *11*, e0154371. https://doi.org/10.1371/journal.pone.0154371.

Ng, Y.S., Bindoff, L.A., Gorman, G.S., Horvath, R., Klopstock, T., Mancuso, M., Martikainen, M.H., Mcfarland, R., Nesbitt, V., Pitceathly, R.D.S., et al. (2019). Consensus-based statements for the management of mitochondrial stroke-like episodes. Wellcome Open Res. *4*, 201. https://doi.org/10.12688/wellcomeopenres.15599.1.

Pek, N.M.Q., Phua, Q.H., Ho, B.X., Pang, J.K.S., Hor, J.H., An, O., Yang, H.H., Yu, Y., Fan, Y., Ng, S.Y., et al. (2019). Mitochondrial 3243A > G mutation confers pro-atherogenic and pro-inflammatory properties in MELAS iPS derived endothelial cells. Cell Death Dis. *10*, 802. https://doi.org/10.1038/s41419-019-2036-9.

Perez-Riverol, Y., Bai, J., Bandla, C., García-Seisdedos, D., Hewapathirana, S., Kamatchinathan, S., Kundu, D.J., Prakash, A., Frericks-Zipper, A., Eisenacher, M., et al. (2022). The PRIDE database resources in 2022: a hub for mass spectrometry-based proteomics evidences. Nucleic Acids Res. *50*. D543–d552. https://doi.org/10.1093/nar/gkab1038.

Picard, M., Zhang, J., Hancock, S., Derbeneva, O., Golhar, R., Golik, P., O'Hearn, S., Levy, S., Potluri, P., Lvova, M., et al. (2014). Progressive increase in mtDNA 3243A>G heteroplasmy causes abrupt transcriptional reprogramming. Proc. Natl. Acad. Sci. USA *111*, E4033–E4042. https://doi.org/10.1073/pnas.1414028111.

Pickett, S.J., Grady, J.P., Ng, Y.S., Gorman, G.S., Schaefer, A.M., Wilson, I.J., Cordell, H.J., Turnbull, D.M., Taylor, R.W., McFarland, R., and McFarland, R. (2018). Phenotypic heterogeneity in m.3243A>G mitochondrial disease: The role of nuclear factors. Ann. Clin. Transl. Neurol. *5*, 333–345. https://doi.org/10.1002/acn3.532.

Qi, Y., Ye, Y., Wang, R., Yu, S., Zhang, Y., Lv, J., Jin, W., Xia, S., Jiang, W., Li, Y., et al. (2022). Mitochondrial dysfunction by TFAM depletion disrupts self-renewal and lineage differentiation of human PSCs by affecting cell proliferation and YAP response. Redox Biol. *50*, 102248. https://doi.org/10.1016/j.redox.2022.102248.

Ryytty, S., and Hämäläinen, R.H. (2023). The Mitochondrial m.3243A>G Mutation on the Dish, Lessons from In Vitro Models. Int. J. Mol. Sci. *24*, 13478. https://doi.org/10.3390/ijms241713478.

Ryytty, S., Modi, S.R., Naumenko, N., Shakirzyanova, A., Rahman, M.O., Vaara, M., Suomalainen, A., Tavi, P., Hämäläinen, R.H., and Hämäläinen, R.H. (2022). Varied Responses to a High m.3243A>G Mutation Load and Respiratory Chain Dysfunction in Patient-Derived Cardiomyocytes. Cells *11*, 2593. https://doi.org/10.3390/cells11162593.

Shen, X., and Du, A. (2021). The non-syndromic clinical spectrums of mtDNA 3243A>G mutation. Neurosciences *26*, 128–133. https://doi.org/10.17712/nsj.2021.2.20200145.

Silva-Pinheiro, P., and Minczuk, M. (2022). The potential of mitochondrial genome engineering. Nat. Rev. Genet. *23*, 199–214. https://doi.org/10.1038/s41576-021-00432-x.

Sin, J., Andres, A.M., Taylor, D.J.R., Weston, T., Hiraumi, Y., Stotland, A., Kim, B.J., Huang, C., Doran, K.S., Gottlieb, R.A., and Gottlieb, R.A. (2016). Mitophagy is required for mitochondrial biogenesis and myogenic differentiation of C2C12 myoblasts. Autophagy *12*, 369–380. https://doi.org/10.1080/15548627.2015.1115172.

Tang, D., Chen, M., Huang, X., Zhang, G., Zeng, L., Zhang, G., Wu, S., Wang, Y., and Wang, Y. (2023). SRplot: A free online platform for data visualization and graphing. PLoS One *18*, e0294236. https://doi.org/10.1371/journal.pone.0294236.

Tranah, G.J., Katzman, S.M., Lauterjung, K., Yaffe, K., Manini, T.M., Kritchevsky, S., Newman, A.B., Harris, T.B., Cummings, S.R., and Cummings, S.R. (2018). Mitochondrial DNA m.3243A > G heteroplasmy affects multiple aging phenotypes and risk of mortality. Sci. Rep. *8*, 11887. https://doi.org/10.1038/s41598-018-30255-6.

Valdebenito, G.E., Chacko, A.R., and Duchen, M.R. (2023). The mitochondrial ATP synthase as an ATP consumer-a surprising therapeutic target. EMBO J. *42*, e114141. https://doi.org/10.15252/embj.2023114141.

Yokota, M., Hatakeyama, H., Ono, Y., Kanazawa, M., and Goto, Y.I. (2017). Mitochondrial respiratory dysfunction disturbs neuronal and cardiac lineage commitment of human iPSCs. Cell Death Dis. *8*, e2551. https://doi.org/10.1038/cddis.2016.484.

Stem Cell Reports, Volume *20*

Supplemental Information

Metabolic remodeling in hiPSC-derived myofibers carrying the m.3243A>G mutation

Gabriel E. Valdebenito, Anitta R. Chacko, Chih-Yao Chung, Preethi Sheshadri, Haoyu Chi, Benjamin O'Callaghan, Monika J. Madej, Henry Houlden, Hannah Rouse, Valle Morales, Katiuscia Bianchi, Francesco Saverio Tedesco, Robert D.S. Pitceathly, and Michael R. Duchen

SUPPLEMENTAL FIGURES

Fig S1

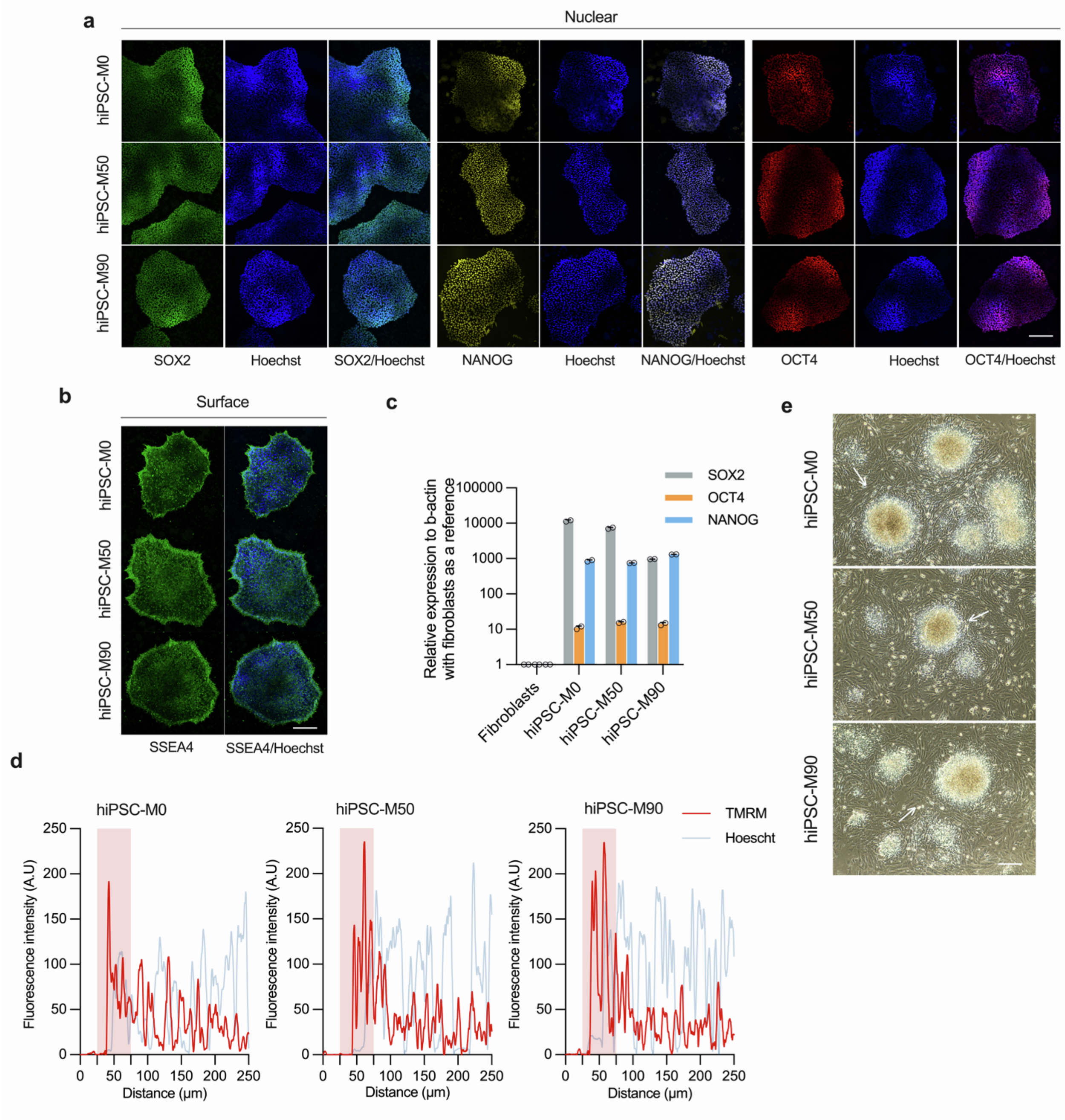

Supplementary Figure 1. Maintenance of pluripotency and mitochondrial function during the undifferentiated stage of hiPS cells.

(A) Representative images of hiPS cell colonies stained for nuclear pluripotency markers SOX2, NANOG and OCT4. Scale bar = 100 µm.

(B) Representative images of hiPS cell colonies stained for the pluripotency surface marker SSEA4. Scale bar = 100 µm.

(C) Relative gene expression of pluripotency markers thought RT-qPCR (*n* = 2 independent biological samples).

(D) Cross sectional fluorescence quantification of hiPSC colonies loaded with 25 nM TMRM. Coloured background represents 50 µM from the border of the colony to an arbitrary centre.

(E) Bright-field images of differentiating cells depicting the formation of myocentres. Arrows indicate the myocentres. Scale bar = 100 µm.

Source data are provided as a Source Data file. All data were represented as mean ± SD.

Fig S2

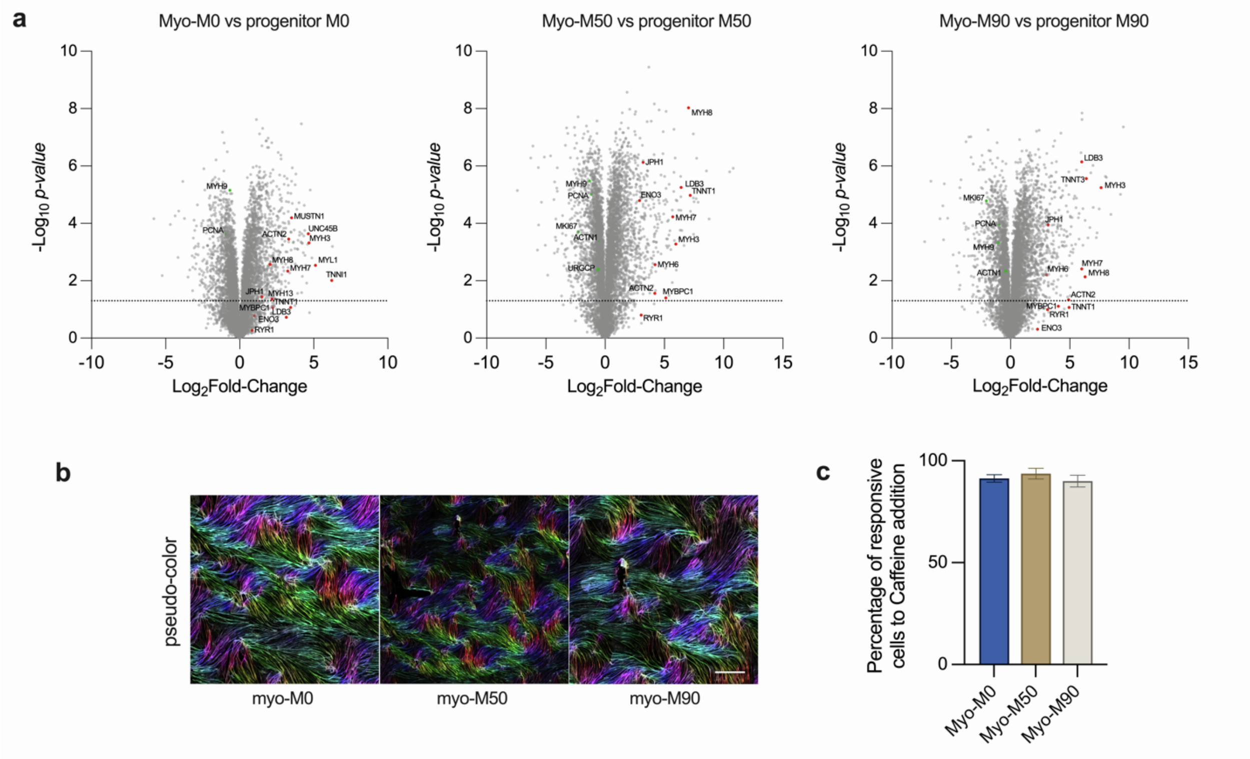

Supplementary Figure 2. Changes in the proteome after differentiation, bundle formation and calcium responses in myofibres derived from hiPSCs.

(A) Volcano plot showing changes in the proteome. Changes are expressed as myofibre over its respective progenitor for each line. Genes above the dotted line express significant changes (-Log p-value ≥ 1.3). Red dots = upregulated proteins; green dots = downregulated proteins.

(B) Representative confocal images of myofibres stained with antibodies against alpha-actinin 2 and pseudo-coloured in relation to the fibre orientation. Scale bar = 500 µm.

(C) Quantification of responsive myofibres to caffeine addition. Arbitrary threshold of 10% change over basal intensity was defined to consider a cell as responsive. n > 100 myofibres were analysed per condition.

Source data are provided as a Source Data file. All data were represented as mean ± SD and data were analysed by one-way ANOVA with Tukey's multiple comparisons test (*p < 0.05, **p < 0.01, ***p < 0.001, ****p < 0.

0001).

Fig S3

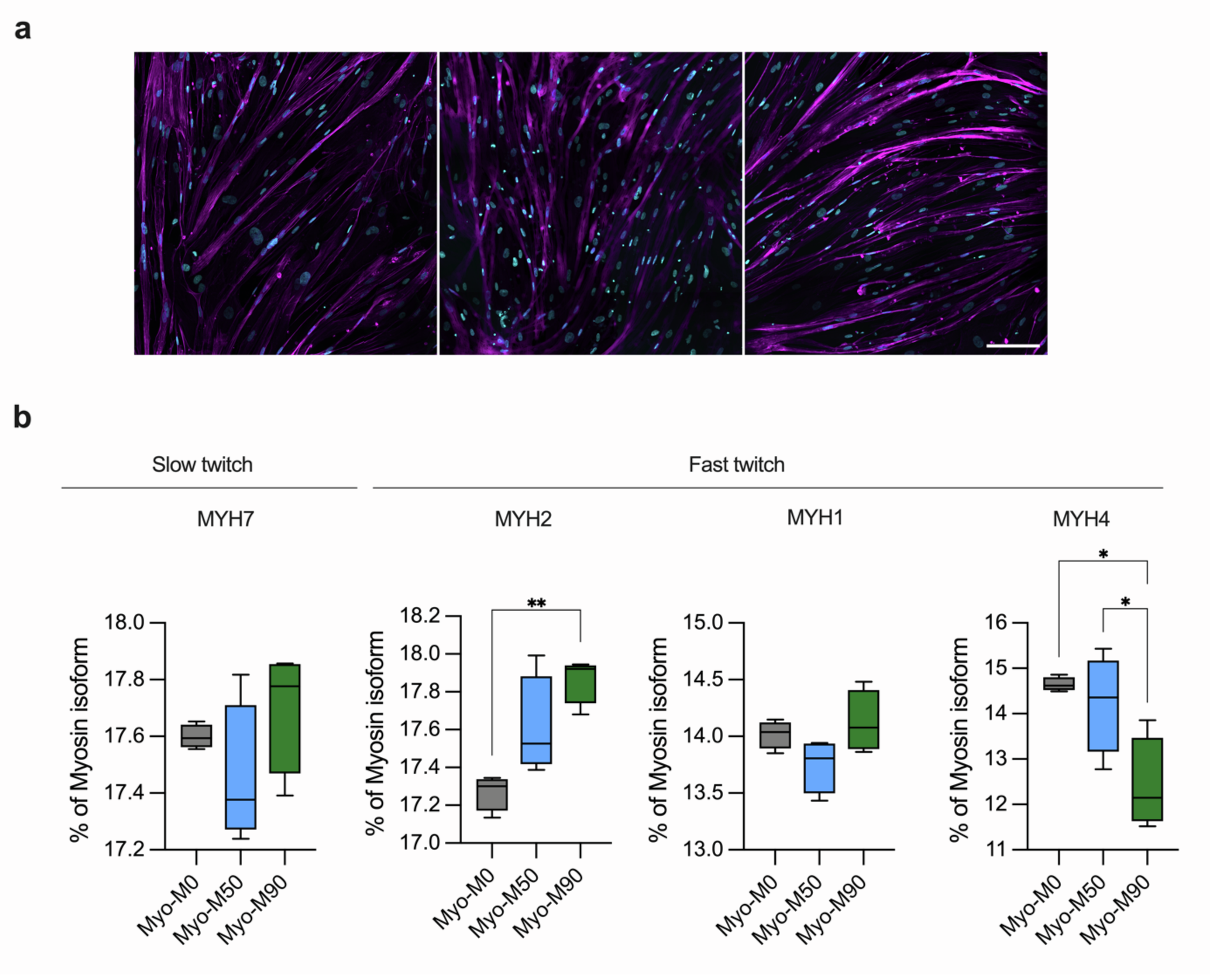

Supplementary Figure 3. Fiber distribution in myofibres expressing the m.3243A>G

(A) Representative images used to quantify nuclei per cell in Figure 3A.

(B) Comparison of the protein abundance obtained from the proteomic analysis. $n = 3$ independent replicates.

Source data are provided as a Source Data file. All data were represented as mean ± SD and data were analysed by one-way ANOVA with Tukey's multiple comparisons test (*p < 0.05, **p < 0.01, ***p < 0.001, ****p < 0.0001).

Fig S4

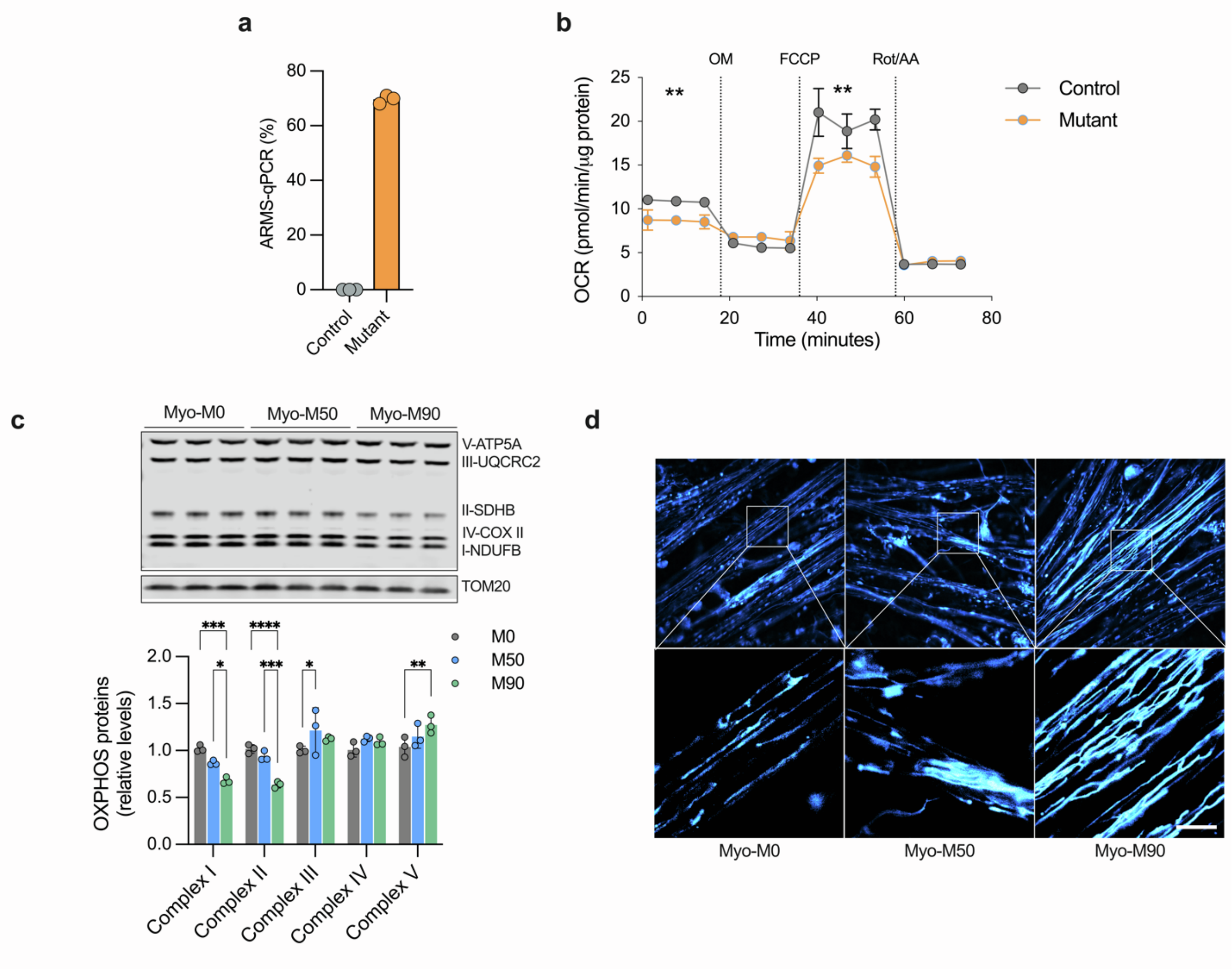

Supplementary Figure 4. Fiber distribution in myofibres expressing the m.3243A>G.

(A) ARMS-qPCR of unrelated control and mutant lines expressing the m.3243A>G mutation. $n = 3$ independent biological replicates.

(B) Cell respiratory capacity measured using the Seahorse XFe96 extracellular flux analyser in myofibres ($n = 3$, 6 culture wells per experiment).

(C) Protein expression of mitochondrial respiratory complexes subunits ($n = 3$ independent biological replicates). TOM20 was used as loading control. Images are representative of at least three independent experiments.

(D) Representative confocal images of NAD(P)H autofluorescence. Scale bar = 100 µM.

Fig S5

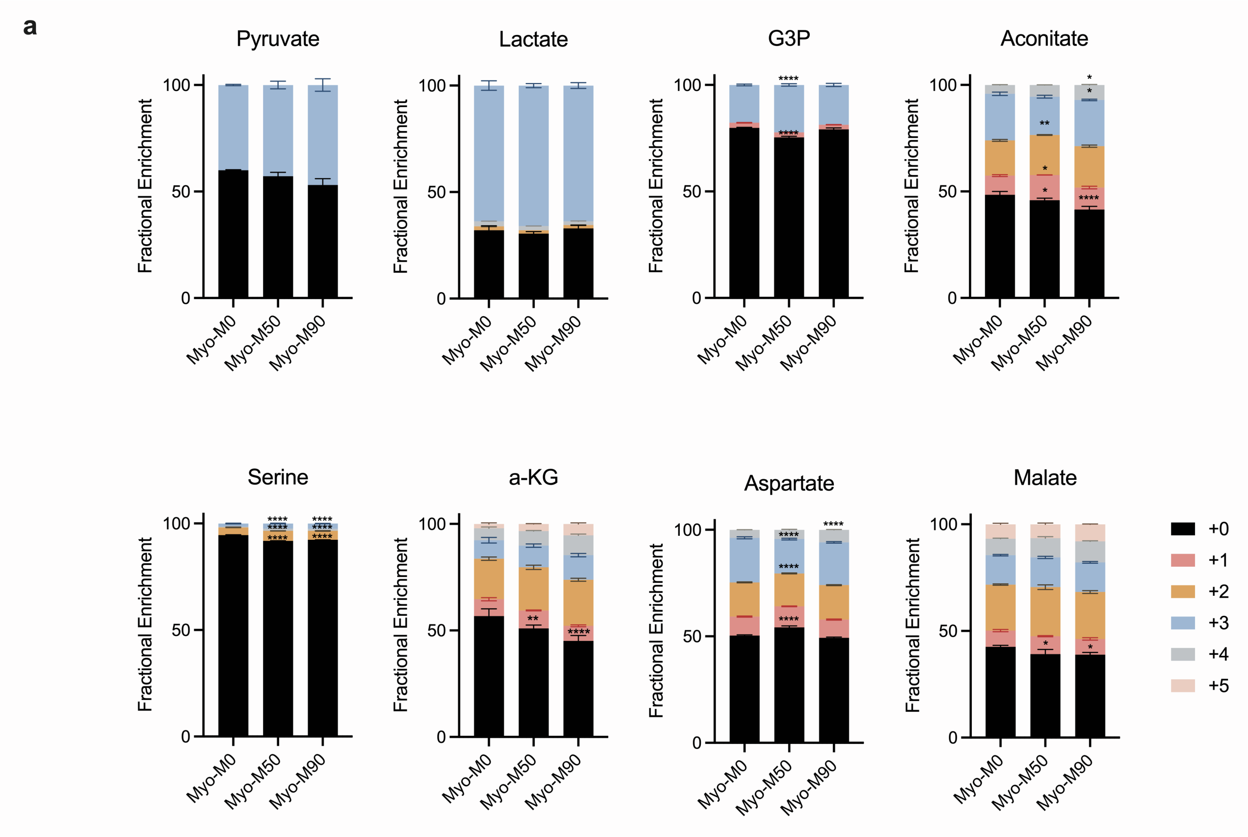

Supplementary Figure 5. Targeted metabolomic analysis showed changes in labelling pattern of metabolites

(A) Fractional enrichment of 13C isotopologues measured by targeted metabolomic (*n* = 3 independent biological replicates).

Source data are provided as a Source Data file. All data were represented as mean ± SD and data were analysed by one-way ANOVA with Tukey's multiple comparisons test (*p < 0.05, **p < 0.01, ***p < 0.001, ****p < 0.0001).

SUPPLEMENTAL METHODS

The mitochondrial oxygen consumption rate

Measurements of oxygen consumption were conducted with the Seahorse Bioscience XFe96 bioanalyzer using the Seahorse XF Cell Mito Stress Test Kit (Cat# 103015-100, Agilent). hiPSC and progenitors were seeded on XF96 cell culture microplates (Cat# 102416-100, Agilent). On the day of the experiment, the culture medium was replaced with Seahorse XF Base medium (Cat# 103334-100, Agilent) supplemented with 1 mM pyruvate (Cat# 11360070, Gibco), 2 mM glutamine (Cat# 25030081, Gibco) and 10 mM glucose (Cat# A2494001, Gibco) and incubated for 30 min at 37 °C in a CO_2-free incubator before loading into the Seahorse Analyser. After measuring basal respiration, the drugs oligomycin (1 µM), FCCP (1 µM, 2 µM), and rotenone/antimycin A (0.5 µM/0.5 µM) were added to each well in sequential order. Data were analysed using the XF Cell Mito Stress Test Report Generator. After the assay, protein was extracted from each well and a BCA assay was performed. The normalisation of the experiments is based on the relative protein obtained.

RT–qPCR

RNA was extracted using the Qiagen RNAeasy Kit (Cat# 74104, Qiangen) following the manufacturer's instructions. RNA concentration was quantified by measuring 260 nm absorbance using a nanodrop spectrophotometer. A complementary DNA (cDNA) library was immediately created from 500 ng of the RNA in a reverse transcription (RT) reaction using the SuperScript II (SSII) Reverse Transcriptase (Cat# 18064014, ThermoFisher). Reverse transcription was conducted in accordance with the Applied Biosystems guidelines. The obtained cDNA was then diluted 1:30 in H_2O in order to maintain an appropriate cycle threshold (Ct) range. Gene expression analysis employed SYBR Green JumpStart Taq ReadyMix (Cat# S4438, Sigma-Aldrich) on the CFX-Connect RT–PCR System, utilising CFX Manager Software version 2.1 (Bio-Rad) as per the manufacturer's instructions. The qPCR data were analysed using the delta-delta Ct method. Primer sequences used are specified as follow:

Target	Forward primer	Reverse primer
OCT4	GACAGGGGGAGGGGAGGAGCTAGG	CTTCCCTCCAACCAGTTGCCCCAAAC
SOX2	GGGAAATGGGAGGGGTGCAAAAGAGG	TTGCGTGAGTGTGGATGGGATTGGTG
NANOG	TGCAAGAACTCTCCAACATCCT	ATTGCTATTCTTCGGCCAGTT
ACTB (housekeeping)	CACCATTGGCAATGAGCGGTTC	AGGTCTTTGCGGATGTCCACGT

Western blot

Myofibres were washed with ice-cold PBS once and lysed using 100 µl RIPA buffer (Cat# R0278, Sigma-Aldrich) with Protease and Phosphatase Inhibitor Cocktail (Cat# 78440, ThermoFisher). Cells were then scraped and stored at -80 C. Protein concentration was quantified using the Pierce BCA Assay Kit (Cat# 23227, ThermoFisher). For immunoblotting, 30 µg of protein samples in NuPAGE 4x LDS Sample Buffer (Cat# NP0007, Invitrogen) and 2% β-mercaptoethanol (Cat# 63689, Sigma-Aldrich) were boiled at 99ºC for 5 min. Proteins were separated on 4-12% NuPAGE Bis-Tris polyacrylamide gels (Cat# NP0335, Invitrogen) and transferred onto PVDF membranes (Cat# IPFL00010, Millipore). Membranes were then incubated in Intercept (TBS) Blocking Buffer (Cat# 927-60001, Li-COR Biosciences) for 1 h at room temperature. After addition of primary antibodies diluted in the blocking buffer with 0.1% Tween-20, membranes were incubated overnight at 4ºC on a shaker. Subsequently, membranes were incubated with appropriate secondary antibodies (Li-COR Biosciences; 1:10000; IRDye® 680RD Goat anti-Mouse IgG, #926-68070; IRDye® 800CW Goat anti-Rabbit IgG, #926-32211) for 1 h at room temperature before signals were developed with the LiCOR Odyssey CLx system.

**Antibodies**	**#Cat**	**Provider**	**Dilution**
alpha Actinin 2 Monoclonal Antibody (EA-53)	MA5-49311	ThermoFisher	1:250
Myosin 4 Monoclonal Antibody (MF20), eBioscience™	14-6503-82	ThermoFisher	1:100
OxPhos Human WB Antibody Cocktail	45-8199	ThermoFisher	1:1000
GPD1 Polyclonal antibody	27943-1-AP	Proteintech	1:1000
GPD1L Polyclonal antibody	17263-1-AP	Proteintech	1:1000

GPD2 Polyclonal antibody	17219-1-AP	Proteintech	1:1000
MDH1 Polyclonal antibody	15904-1-AP	Proteintech	1:1000
Anti-beta Actin antibody	ab8227	abcam	1:1000
Anti-p-S6	4858	Cell Signaling Technology	1:1000
Anti-S6	2217	Cell Signaling Technology	1:1000
Anti-p-Akt	9271	Cell Signaling Technology	1:500
Anti-Akt	9272	Cell Signaling Technology	1:1000

Immunofluorescence

Pluripotent stem cells were seeded at single-cell colonies with ROCK inhibitor (Cat# 1254/10, Bio-techne) for one day. Then, cells were washed, and the media was changed daily until colonies appeared in the culture well. The cells were washed three times with 1X PBS (Cat# 14190144, ThermoFisher) and fixed in 4% paraformaldehyde for 15 min at room temperature and permeabilized with 0.1% Triton X-100 (Cat# 85111, ThermoFisher) for 30 min in PBS. The cells were then washed and incubated with primary antibodies (Myosin Skeletal Muscle antibody, MA1-90701; ACTN2 antibody, 14221-1-AP) in 3% BSA overnight at 4 degrees followed by incubation with Alexa Fluor-conjugated secondary antibodies for 1 h at room temperature. After antibody labelling, the coverslips were mounted on a glass slide using ProLong™ Gold Antifade Mounting (Cat# P36930, ThermoFisher) with DAPI and imaged using the confocal microscope as described above. Image post-processing was performed in ImageJ/FIJI.

Spatial organisation of neighbouring myofibers

Progenitors were differentiated in 35 mm glass-bottom dishes. On the day of the experiment, cells were washed twice with PBS (Cat# 14190144, ThermoFisher) and then fixed with 4% PFA. To observe the directionality of the myofibers in culture, the cells were incubated with α-actinin 2 antibody, followed by Alexa Fluor-conjugated secondary antibody incubation. Images were acquired using Zen Black software

(Carl Zeiss) as described above. Then, images were pseudo-coloured and anal according to Mao et al, 2022.

Lentivirus production

Peredox NADH/NAD^+ sensor (Cat# 163060, Addgene) was transfected into HEK293 cells for lentivirus production. Briefly, the cells were seeded at 70-80% confluency in 10 cm dishes the day before transfection. The constructs (1.2 µg) were co-transfected with vesicular stomatitis virus G (600 ng) and pSPAX2 (800 ng) using Lipofectamine 3000 (Cat# L3000001, ThermoFisher). The lentiviral supernatants were collected 2 days after transfection, and then the cleared supernatant was concentrated with Lenti-X Concentrator (Cat# 631232, Takara) and resuspended in 1 mL of DMEM.

The viral suspension was then added to the pluripotent stem cells as described by Diaz-Cuadros et al. (2023). Blasticidin (1 µg/mL) was used to select cells with the integrated construct. Lentivirus transduction was evaluated using a CLARIOstar microplate reader (excitation/emission = 580/610 nm for mCherry, and excitation/emission = 400/513 nm for Peredox).

Medium pH values

Medium pH values were measured based on the ratiometric property of phenol red. Progenitors were grown in 96-well plates with 150 µl of differentiation media and cultured for 5 days. On day 5, the media was replaced, and samples were collected on day 10. On the day of the experiments, the media from each well was transferred to a new 96-well plate, and the absorbance of phenol red at 443 and 570 nm was immediately measured. The higher the absorbance ratios of 443 to 570 nm, the more acidic the media. A BCA assay was done to ensure the same amount of cells/proteins were obtained per culture well.

Targeted metabolomics

Progenitors were seeded in 60 mm dishes and differentiated for 10 days. Myofibres were then cultured in medium containing 5 mM $^{13}C_6$-glucose (Cat# CLM-1396-5, Cambridge Isotope Laboratories) for an additional 18 h before metabolite isolation. Briefly, cells were washed with PBS (Cat# 14190144, ThermoFisher) three times and resuspended in ice-cold extraction buffer (20% ultrapure water, 50% methanol, 30% acetonitrile) at a ratio of 20 × 10^6 cells per ml. Subsequently, the cells were incubated on methanol and dry ice for 15 min, placed on a shaker for an additional 15 min at 4 °C, and then at –20 °C for 1 h. The cell lysate was centrifuged, and the supernatant was collected and transferred to autosampler

glass vials, which were stored at –80 °C. LC-MS analysis was performed using a Q Exactive Quadrupole-Orbitrap mass spectrometer coupled to a Vanquish UHPLC system (Thermo Fisher Scientific). The liquid chromatography system was fitted with a Sequant ZIC-pHILIC column (150 mm × 2.1 mm) and guard column (20 mm × 2.1 mm) from Merck Millipore (Germany) and temperature maintained at 35°C. The sample (3 μL) was separated at a flow rate of 0.1 mL/min. The mobile phase was composed of 10 mM ammonium bicarbonate and 0.15% ammonium hydroxide in water (solvent A), and acetonitrile (solvent B). A linear gradient was applied by increasing the concentration of A from 20 to 80% within 22 min and then maintained for 7 minutes. The mass spectrometer was operated in full MS and polarity switching mode, in the range of 70-1000m/z and resolution 70000. Major ESI source settings were: spray voltage 3.5 kv, capillary temperature 275°C, sheath gas 35, auxiliary gas 5, AGC target 3e6, and maximum injection time 200 ms. For the targeted analysis, the acquired spectra were analysed using XCalibur Qual Browser and XCalibur Quan Browser software (Thermo Scientific).

NAD(P)H autofluorescence

Progenitors were differentiated in 35 mm glass-bottom dishes. On the day of experiments, cells were washed twice with PBS (Cat# 14190144, ThermoFisher) and then incubated in recording media (DMEM no phenol red). Images were captured on a Zeiss 880 confocal microscope, equipped with a 60x UV-VIS oil immersion objective at 37 °C with excitation at 355 nm. Images were acquired under basal conditions, and after the addition of NaCN to a final concentration of 1 μM and FCCP at a final concentration of 1 μM. A wash with PBS (Cat# 14190144, ThermoFisher) was performed between the drug additions, and fresh media were replenished into the culture wells. The images were analysed with Fiji, and the quantification was done following a previous publication (Chi, Bhosale, & Duchen, 2022).
